# *N* ^6^-Methyladenine hinders RNA- and DNA-directed DNA synthesis: application in human rRNA methylation analysis of clinical specimens†

**DOI:** 10.1039/c5sc02902c

**Published:** 2015-11-17

**Authors:** Shaoru Wang, Jiaqi Wang, Xiaoe Zhang, Boshi Fu, Yanyan Song, Pei Ma, Kai Gu, Xin Zhou, Xiaolian Zhang, Tian Tian, Xiang Zhou

**Affiliations:** a College of Chemistry and Molecular Sciences, Key Laboratory of Biomedical Polymers of Ministry of Education, The Institute for Advanced Studies, Wuhan University Wuhan Hubei 430072 P. R. China xzhou@whu.edu.cn ttian@whu.edu.cn +86-27-68756663 +86-27-68756663; b Zhongnan Hospital, Wuhan University Wuhan 430071 Hubei Province China; c School of Medicine, Wuhan University Wuhan 430071 China

## Abstract

*N* ^6^-Methyladenine (m^6^A) is the most abundant internal modification on mammalian mRNA. Very recently, m^6^A has been reported as a potentially important ‘epigenetic’ mark in eukaryotes. Until now, site-specific detection of m^6^A is technically very challenging. Here, we first reveal that m^6^A significantly hinders DNA- and RNA-directed DNA synthesis. Systematic investigations of 5′-triphosphates of a variety of 5-substituted 2′-deoxyuridine analogs in primer extension have been performed. In the current study, a quantitative analysis of m^6^A in the RNA or DNA context has been achieved, using *Bst* DNA polymerase catalyzed primer extension. Molecular dynamics study predicted that m^6^A in template tends to enter into and be restrained in the MGR region of *Bst* DNA polymerase, reducing conformational flexibility of the DNA backbone. More importantly, a site-specific determination of m^6^A in human ribosomal RNA (rRNA) with high accuracy has been afforded. Through a cumulative analysis of methylation alterations, we first reveal that significantly cancer-related changes in human rRNA methylation were present in patients with hepatocellular carcinoma.

## Abstract 2

Here, we report that m^6^A significantly hinders DNA- and RNA-directed DNA synthesis, and a quantitative analysis of m^6^A in RNA or DNA context has been achieved..

## Graphical abstract

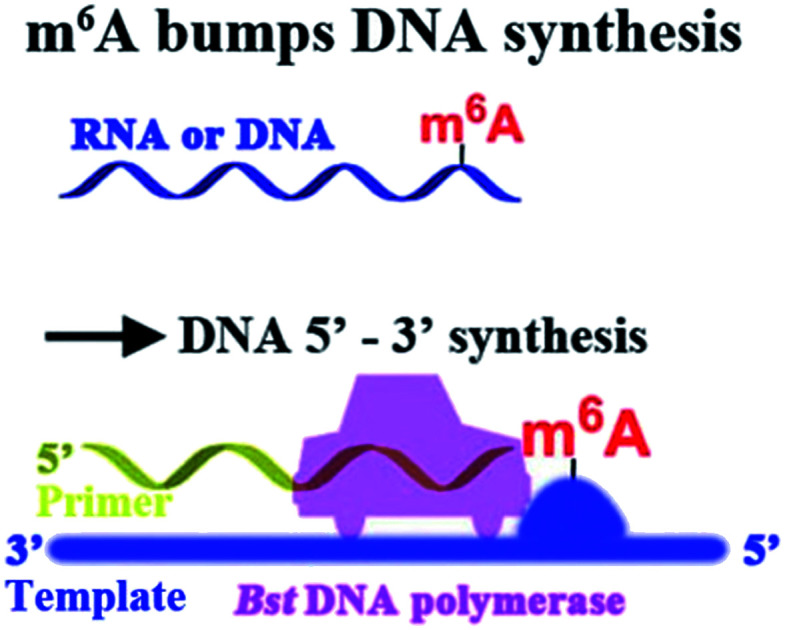

## Introduction

**Fig. 1:**
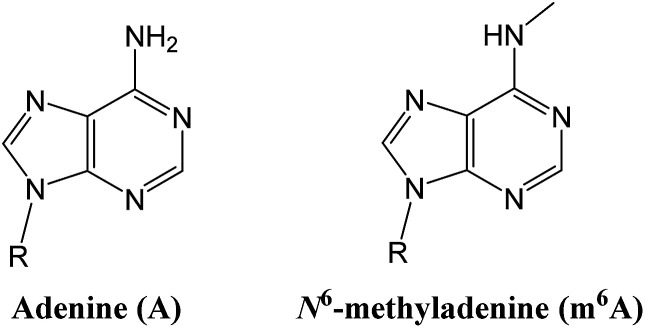
Structure illustration of adenine and *N*^6^-methyladenine (m^6^A).

Epigenetic modifications on nucleic acids can significantly regulate the long-term gene activity and expression without any alteration in nucleotide sequence.^1^ Both DNA and RNA can be methylated at the *N*^6^ position of adenine (‘A’ in Fig. 1) to form *N*^6^-methyladenine (‘m^6^A’ in Fig. 1),^2^ which is one of the most important and common epigenetic markers. m^6^A is a prevalent modification present in the genome of bacteria^3^ and plays importantly regulatory roles in DNA restriction-modification systems.^4^ More importantly, several key reports have suggested that it may have a gene regulatory function in eukaryotes, including green alga,^5^ worm,^6^ and fly.^7^ Since the discovery of m^6^A demethylation in mammalian mRNAs by fat mass and obesity associated protein (FTO)^8^ and ALKBH5,^9^ there has been a great burst of interest in RNA epigenetics study.^10^

Due to the vital roles of m^6^A, dysregulation of RNA methylation can be associated with aberrant gene expression, which further lead to human diseases.^11^ In particular, some studies have shown that obesity and related diseases (type II diabetes) are associated to increased FTO activity and abnormally decreased amounts of m^6^A in patients.^12^ To the best of our knowledge, human cancers almost universally develop dysregulation of epigenetic marks, during both cancer initiation and disease progression.^13^ Since total RNA can be easily isolated from samples derived from cancer patients, it is well suited for further detection.^14^ Until now, the association of m^6^A modification with human cancer remains elusive, probably due to limited detection strategies. Recently, a pioneering method for m^6^A determination has been developed using a recombinant *Thermus thermophilus* DNA polymerase I (*Tth* pol) and specific m^6^A residues have been determined in human RNAs.^15^ However, the polymerization activities of *Tth* pol in RNA-directed DNA synthesis still remains to be improved.

**Fig. 2:**
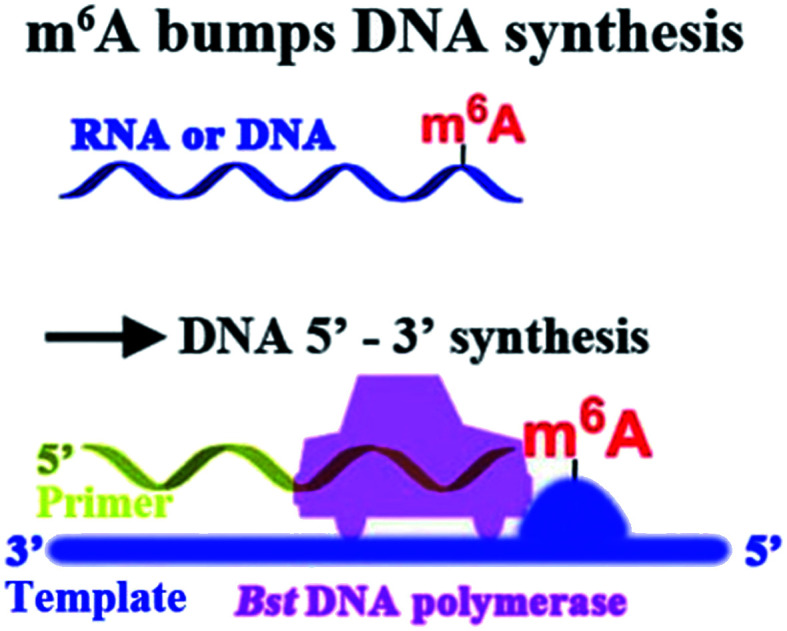
m^6^A hinders DNA- or RNA-directed DNA synthesis.

Here, we report that m^6^A significantly hinders DNA- and RNA-directed DNA synthesis (Fig. 2) using a different polymerase, and a quantitative analysis of m^6^A in RNA or DNA context has been achieved. As an application of our approach, a site-specific determination of m^6^A in human ribosomal RNA (rRNA) of cultured tumor cells has been afforded. More importantly, different levels of methylation in human rRNA between hepatic cancerous and paracancerous tissues of patients with hepatocellular carcinoma have been identified using the new strategy. Our findings may provide new insights on DNA and RNA epigenetics of m^6^A.

## Results

### *N* ^6^-Methyladenine hinders DNA-dependent DNA synthesis

Since m^6^A in DNA may play an important regulatory role, we first conducted a study to illustrate its effects on DNA-directed DNA synthesis. *Bst* DNA polymerase, large fragment (*Bst*) is commercially available and is widely used in molecular biology applications.^16^ Here, it is used in the current study using DNA templates containing a site-specific A or m^6^A.

**Fig. 3:**
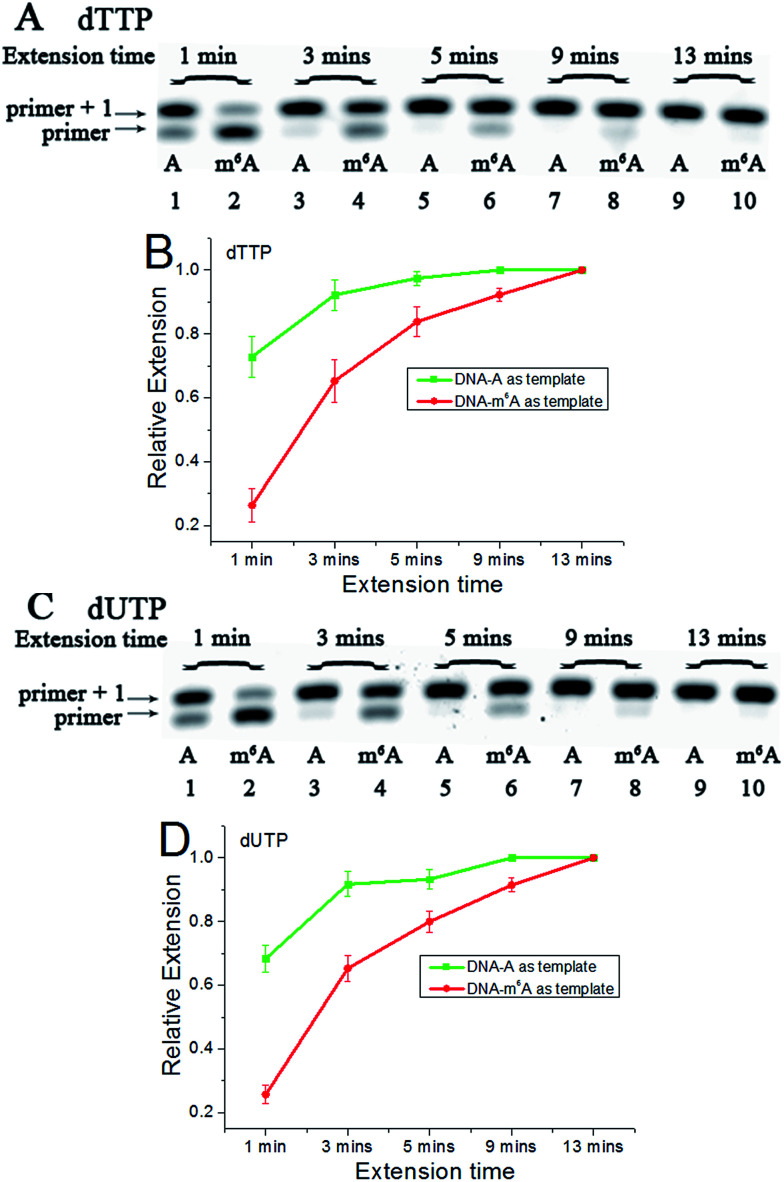
The analysis displays the difference of nucleotide incorporation opposite a templating A or m^6^A. Lane 1, 3, 5, 7, 9, DNA-A was used as a template; lane 2, 4, 6, 8, 10, DNA-m^6^A was used as a template. (A) and (C), representative gel image showing incorporation of dTTP or dUTP; (B) and (D), all data are presented as the means ± SEM from three independent experiments.

DNA-A, DNA-m^6^A and primer 1 (sequences in Table S1†) were used to set up the model reaction. The dTTP or dUTP (structures in Fig. S1†) was used, and the incorporation efficiencies following different incubation times for DNA-A or DNA-m^6^A were compared together. In the current study, parameter RE (relative extension value) refers to normalized value of amount of reacted primer (‘primer + 1’ product) relative to the total amount of primer DNA. As shown in Fig. 3, a consistently lower RE for dTTP and dUTP opposite the m^6^A-template was characterized. For the same strand (Fig. S2†), a similar RE for dTTP incorporation compared with dUTP was observed through a same incubation time. Since there is a 5-substituted methyl difference between dUTP and dTTP, it raises an intriguing question about whether new uridine derivatives may affect DNA replication dynamics and enlarge incorporation discrepancies between DNA-A and DNA-m^6^A.

Next, we used 5-hmdUTP and 5-formyl-dUTP (structures in Fig. S1†).^17^ The results demonstrated much lower incorporation efficiencies of both these two triphosphates opposite the DNA-m^6^A compared to DNA-A (Fig. S3†). Also, 5-hmdUTP and 5-formyl-dUTP were incorporated in the extended DNAs with a lower efficiency, compared with dTTP and dUTP (Fig. S4†). Inspired by the electron density sequence of 5-formyl-dUTP < 5-hmdUTP < dTTP, we hypothesised that electron-withdrawing groups would reduce the interaction between the incoming nucleotide and the opposite A or m^6^A.

Subsequently, a family of 5-halogenated uridine analogs, including 5-F-dUTP, 5-Br-dUTP and 5-Iodo-dUTP (structures in Fig. S1†) were used.^18^ As shown in Fig. S5 and S6,† a consistently lower incorporation of 5-halogenated dUTPs opposite DNA-m^6^A was characterized, compared with DNA-A. A comparable amount of 5-Br-dUTP and 5-Iodo-dUTP to dTTP was incorporated into the primer, while much less incorporation of 5-F-dUTP was observed (Fig. S6†).

We further assessed some other sequences to test the universality of our finding. For *Mycobacterium tuberculosis* (*Mtb*), *corA* gene is a strongest candidate methylation-affected gene,^19^ which is methylated at the nucleotide three base pairs downstream of the predicted sigma factor −10 site on the non-template strand while the last nucleotide of the −10 site is methylated on the template strand (Fig. S7†).^19*b*^ Hence, the non-methylated sequence from −9 bp to +15 bp on the non-template strand and the one from −23 bp to −4 bp on the template strand relative to the transcriptional start site (TSS) and their methylated counterparts were synthesized and used as templates for methylation analysis (*corA*-non-A, *corA*-non-m^6^A, *corA*-temp-A and *corA*-temp-m^6^A, sequences in Table S1†). Respective FAM-labelled primers were designed and used in the following study (primer-non and primer-temp in Table S1†). As expected, a much lower incorporation of dTTP or dUTP opposite the m^6^A-templates was characterized, compared with their A-counterparts (Fig. S8 and S9†). The results further suggest that m^6^A hinders DNA-directed DNA synthesis in a sequence-independent manner.

To further evaluate the incorporation efficiency of the studied triphosphates between different templates, we next investigated other DNA polymerases, including Klenow fragment (*exo*-) and Klenow fragment with exonuclease activity. The following results showed that the reduction of substrate activity by adenine methylation was universal (Fig. S10 and S11†), while these two polymerases produced a decreased discrepancy compared with *Bst* DNA polymerase. Hence, *Bst* DNA polymerase was used for further m^6^A analysis in the following studies.

### Calculations studies

**Fig. 4:**
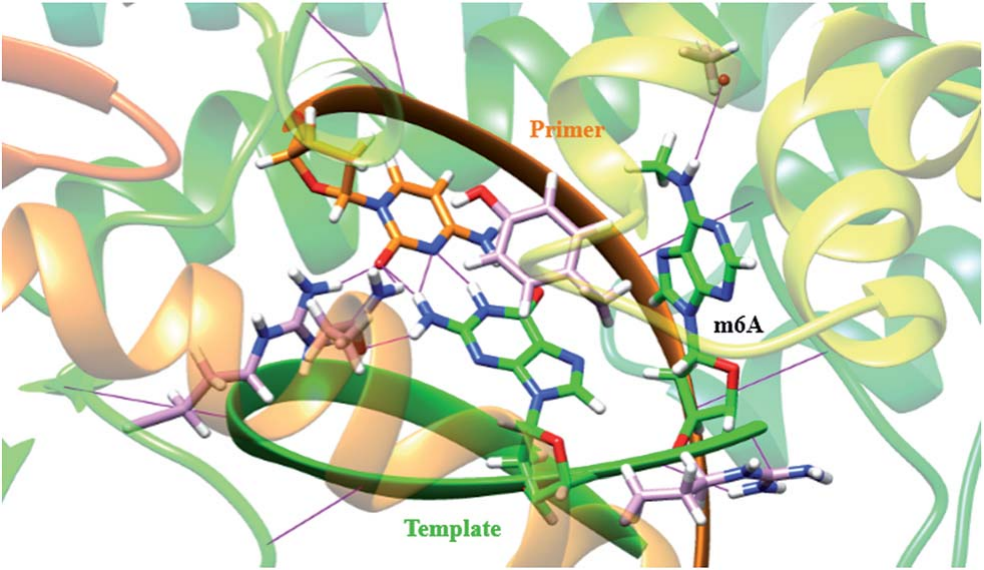
A template containing m^6^A forms a stable complex with primer in the *Bst* DNA polymerase active site place. The structure is superposed with the template–primer (green/orange) duplex. Key residues in the MGR region of enzyme (pink) are displayed, including Tyr 714 (766), Arg 615 (668) and Gln 797 (849). The protein–DNA interface in the MGR region is stabilized by hydrogen bonds (H-bonds, violet) and the stacking interaction between the template base and the corresponding residue [Tyr 714 (766)]. The torsion angles were significantly changed by m^6^A in the template.

In a very relevant study,^20^ Kool and co-workers reported the destabilizing effects of m^6^A in duplexes through NMR analysis and thermodynamic measurements. To gain more insights on structural basis, we investigated the conformation of m^6^A or A in complexes between DNA templates and *Bst* DNA polymerase, using molecular dynamics (MD) approach supplemented with potential of mean force (PMF) analysis.^21^ The starting structure was generated using the deposited crystal structure (PDB code 2BDP), which contains *Bst* DNA polymerase, a primer and a template.^22^ m^6^A was built using DFT (density functional theory) calculation at the B3LYP/6-311G level.^23^ The A that lies in the active site is manually modified to m^6^A. Since the minor-groove recognition (MGR) region in *Bst* DNA polymerase plays a key role to specifically recognize correctly paired bases, it is required to provide sequence-independent interactions with its DNA substrate.^22^ The most common B-DNA conformation is observed for the DNA outside the MGR region, corresponding to the characteristic C2′-*endo* sugar pucker. A pronounced bend in conformation take place at the star of the MGR region (Fig. S12†).^22^ The sugar pucker switches to the C3′-*endo* conformation characteristic of A-form DNA, during the replication process. However, the methyl group of m^6^A significantly perturbates the backbone torsion angles and sugar puckering (detailed torsional angles in Table S2, S3 and Fig. S13†). As demonstrated in Fig. 4, the MD trajectory shows that m^6^A tends to adopt a stubborn B-form with the characteristic C2′-*endo* sugar pucker. Hence, m^6^A in template tends to enter into and be restrained in the MGR region, reducing conformational flexibility for the DNA backbone. This would hamper the essential shift between active sites of *Bst* DNA polymerase and primer–template complex. As a result, it is easier to incorporate dTTP into the growing chain opposing A. Our calculation is therefore consistent to some extent with the previous study.^20^

### Quantitating m^6^A in DNAs

Next, we asked whether our method could be optimized to provide quantitative analysis of m^6^A in DNAs. Therefore, we used 5-hmdUTP and explicitly studied the alteration of the thermal extension times, enzyme concentrations, as well as the extension temperatures, to achieve a better discrimination (Fig. S15†). With the optimized conditions in hand, we mixed known ratios of DNA-m^6^A with the A-containing counterpart (Table S4†) and measured the yield of 5-hmdUTP incorporation at a fixed time point. The values of RE in this reaction were inversely linearly proportional to the amount of m^6^A present (Fig. S16†), suggesting that this method can be used in quantitative analysis of the methylation extent at the candidate site. As shown in Fig. S17,† 5-formyl-dUTP was also successfully used by this method for methylation analysis of DNAs.

### *N* ^6^-Methyladenine hinders RNA-dependent DNA synthesis

**Fig. 5:**
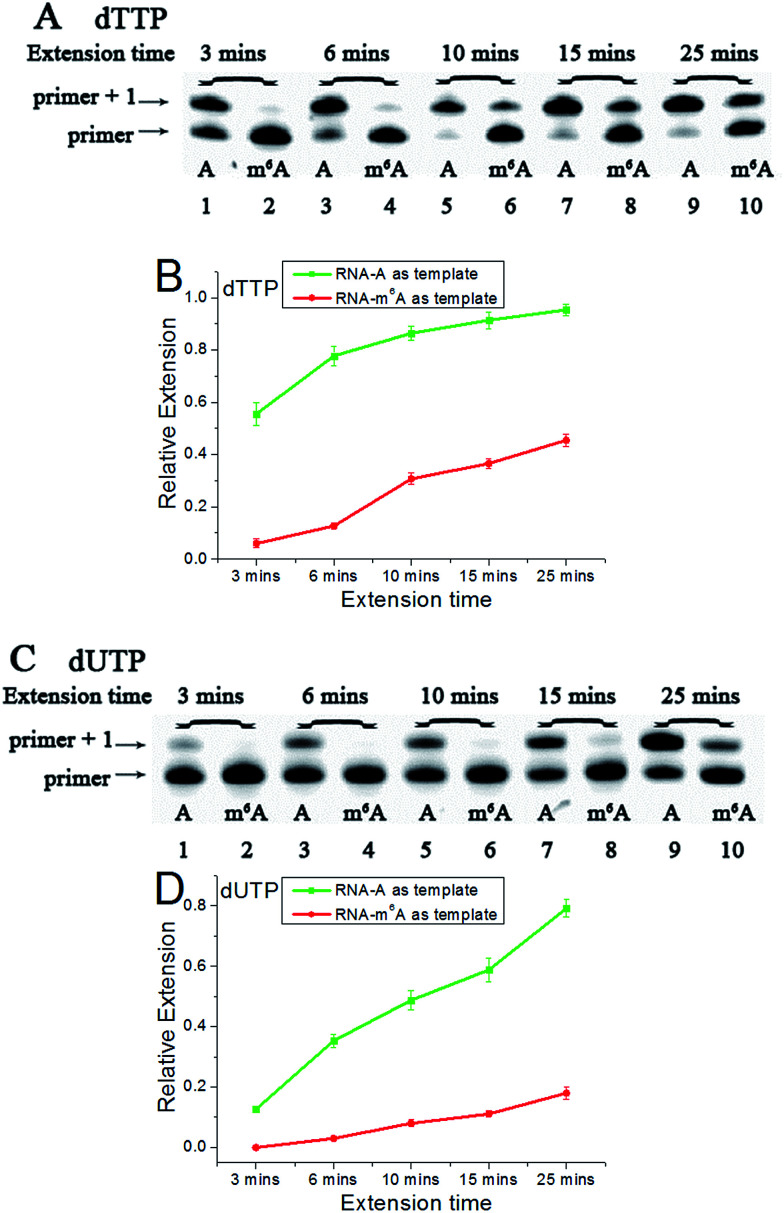
The analysis displays the difference of nucleotide incorporation opposite a templating A or m^6^A. Lane 1, 3, 5, 7, 9, RNA-A used as a template; lane 2, 4, 6, 8, 10, RNA-m^6^A used as a template. (A) and (C), representative gel image showing incorporation of dTTP or dUTP; (B) and (D), all data are presented as the means ± SEM from three independent experiments.

Accounting for the importance of m^6^A in mammalian mRNAs,^24^ we were interested whether the extension discrepancy is present between A- and m^6^A-containing RNA templates. RNA-A, RNA-m^6^A and primer 2 (sequences in Table S1†) were used to perform this study. Through gel analysis, a consistently m^6^A-dependent inhibition was characterized (Fig. 5). RNA-m^6^A incorporates much less dTTPs or dUTPs than RNA-A. For dTTP, a significant extension could proceed beyond the site complementary to the A in RNA-A after an incubation of 3 min, while a moderate to negligible extension in RNA-m^6^A was characterized. Steady-state incorporation kinetics study revealed that RNA-A (*V*_max_/*K*_m_ = 5.12 ± 0.76) is a much better substrate than RNA-m^6^A (*V*_max_/*K*_m_ = 0.16 ± 0.04) for *Bst* DNA polymerase.^25^ For dUTP, much less extension opposite RNA-A compared with dTTP was observed (Fig. S18†), while RNA-m^6^A was not a good template for dUTP incorporation. The extension of dTTP opposite RNA-A was accomplished after an incubation of 6 min (Fig. 5), while the incorporation of 5-formyl-dUTP or 5-hmdUTP was not very efficient after even 15 min (Fig. S19 and S20†).

Next, three 5-halogenated uridine analogs were also assessed and compared for extension behavior opposite the site of A or m^6^A. As shown in Fig. S21,† distinctive extensions were identified as functions of increasing duration. Like dTTP, 5-Iodo-dUTP or 5-Br-dUTP were efficiently incorporated into the growing chain to generate ‘primer + 1’ products. However, because of the strong electron-withdrawing group at the 5 position of 5-F-dUTP, the resulting extension is relatively minimal, as observed in PAGE analyses demonstrating a lower yield for the reaction with 5-F-dUTP than the reaction with other uridine analogs (Fig. S21A and S21B†). A consistent reactivity sequence with the previous DNA template was characterized (Fig. S22†).

### Quantitating m^6^A in synthetic RNAs

We further performed m^6^A analysis of RNA mixtures. A series of artificial samples (Table S5†) were prepared. The incorporation of 5-Iodo-dUTP as a function of increasing RNA-m^6^A concentration is shown in Fig. S23.† The current study showed a very good concordance between the values of RE and the ratios of m^6^A present (Fig. S23B†), suggesting that this method is well suited for RNA methylation analysis at a specific locus. As shown in Fig. S24,† m^6^A analysis in RNA context was also accomplished using dTTP.

### Accurate determinations of m^6^A sites in human ribosomal RNAs

Next, we asked and set out to test directly whether this strategy could be used to determine the methylation states of different sites in rRNA from human cells. We tested the extracted total RNAs from cultured HeLa cells.^26^ A well-known m^6^A modification identified in human rRNA is located at position 1832 in the 18S subunit, and a neighboring A without any modification at position 1781 has been elucidated in a previous study.^27^ Also, the site at position 4984 remains unmodified in the 28S subunit. The according labeled primers targeting these sites have been used.^15^ Since equal amounts of RNAs have been used in the test, direct comparison of RE values could accurately reflect the methylation level. As shown in Fig. S25,† a large proportion of primer1781A (lane 2) and primer4984A (lane 10) was extended, indicating low methylation status of human rRNAs at these sites. In contrast, almost no extended products was observed with primer1832 mA (lane 4), indicating complete methylation status of human rRNAs at the 1832 site. These results are well consistent with the known methylation states of the studied nucleotides.^15,27^

Next, we proceed to determine the methylation status of more A sites in human rRNAs. It has been reported that there is only one m^6^A between positions 4189 and 4190 in human 28S rRNA, while the other one remains unmodified.^28^ Two primers with a same length were used to target the probed nucleotides.^15^ As shown in Fig. S25,† almost all primer4189 were extended to get longer products in the reaction (lane 6), while no extended products were characterized for primer4190 in this analysis (lane 8). This could be interpreted as that human 28S RNA is predominantly methylated at position 4190, while not at position 4189. Our result was entirely consistent with the previous work by another group.^28*b*^ Analysis of cultured MCF-7 tumor cells revealed a very similar m^6^A modification pattern (Fig. S26†).

### rRNA methylation analysis of clinical specimens

**Fig. 6:**
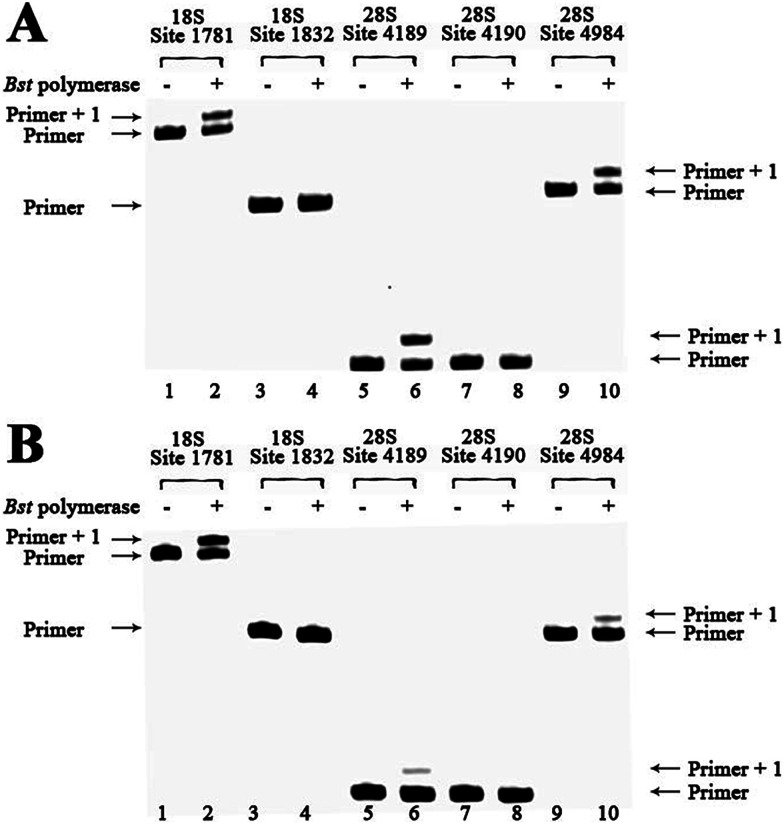
Methylation analysis of 18S and 28S rRNA for hepatic cancerous or paracancerous tissue from a same patient with hepatocellular carcinoma. dTTP is incorporated for analysis. Lanes 1, 3, 5, 7 and 9, control samples without addition of *Bst* DNA polymerase; lanes 2, 4, 6, 8 and 10, 0.1 U *Bst* DNA polymerase was used. (A) Analysis of hepatic cancerous tissues of patient 1 with hepatocellular carcinoma; (B) analysis of hepatic paracancerous tissues of the same patient.

To demonstrate the reliability and make practical application of this method, m^6^A analysis of rRNA in clinical specimens was performed. Fresh hepatic cancerous or paracancerous tissue from a same patient with hepatocellular carcinoma were collected, and total RNAs were extracted using standard methods. The same primers were used to probe the aforementioned sites. Based on these results (Fig. 6 and S27†), human rRNA in both hepatic cancerous and paracancerous tissues was methylated at position 1832 in the 18S subunit and at position 4190 in 28S subunit. However, this quantitative comparative analysis revealed a higher value of RE for hepatic cancerous tissue at positions 4189 and 4984 in 28S subunit, indicating lower levels of RNA methylation at these sites. By contrast, a smaller value of RE for hepatic cancerous tissue at position 1781 in the 18S subunit was observed, indicating a higher level of RNA methylation at this site.

**Fig. 7:**
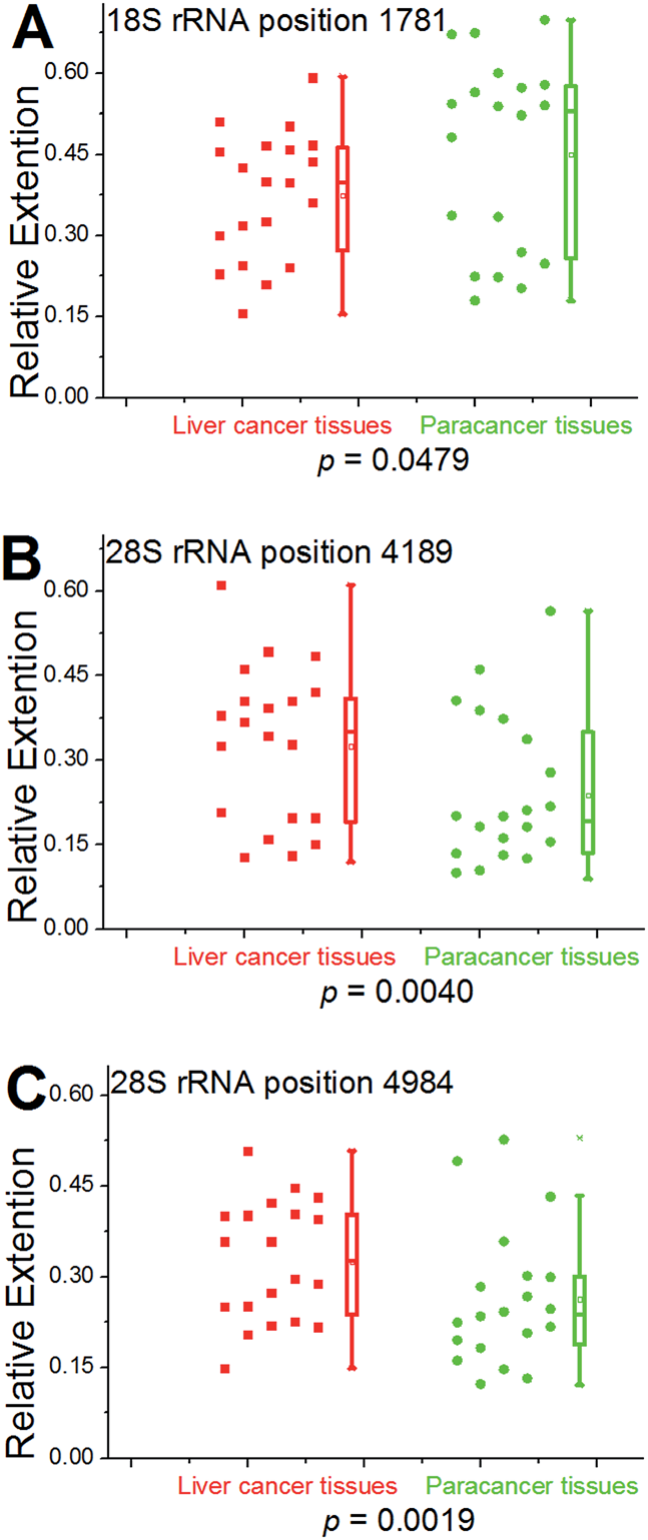
Statistical analysis for 18S and 28S rRNA methylation in clinical specimens. Left scatter plots indicate the RE values of all tested cases obtained from hepatic cancerous or paracancerous tissues in patients with hepatocellular carcinoma. Right box plots represent the distribution of the data. The median value is identified by a line inside the box. The length of the box represents the interquartile range. The *P*-value is calculated by Wilcoxon rank-sum test.

We further used this method to determine rRNA methylation levels at the aforementioned sites within 19 more patients with hepatocellular carcinoma (Fig. S28–S46†). For proof-of-principle, all of the hepatic cancerous tissues from different patients were grouped together and a paracancerous pool was built for comparison. Multivariate statistical analysis was performed to analyze the obtained data using two-sample Hotelling's *T*-squared test. The low *P* value (*P* = 0.0096) provides evidence that there is a significant methylation difference between hepatic cancerous and paracancerous tissues. To further test the methylation difference, Wilcoxon signed-rank test was performed on single locus. As shown in Fig. 7, significantly cancer-related changes were observed in these two pools. For positions 4189 and 4984 (28S RNA), values of RE in hepatic cancerous tissues demonstrate a bimodal distribution, with some having “normal” values (relative to paracancerous tissue) and a second cluster with evidently high values. These results also suggest that the medians and averages of RE at positions 4189 and 4984 (28S RNA) in cancerous tissues are obviously higher than that of the paracancerous control, and while an opposite trend was observed at position 1781 (18S rRNA). Moreover, a bimodal distribution for values of RE was observed in these two groups of tissues, indicating a high heterogeneity of methylation at position 1781 (18S rRNA).

## Discussion

It is very interesting to think about whether m^6^A in an organism can affect central dogma's processes, including DNA replication and RNA reverse-transcription.^29^ In the present study, we first demonstrate that *in vitro* DNA synthesis is hindered by the presence of m^6^A. Recently, m^6^A has been reported to be present in eukaryotic genomic DNAs.^5–7^ Importantly, several m^6^A-binding proteins have been revealed by different groups.^10*c*,30^ As a potentially stable base, m^6^A may affect the folding of chromatin and local replication activity through selective recruitment of m^6^A-binding proteins. Our study therefore proposes a possibility that DNA replication forks may stall at m^6^A sites and such stalling may help the cell avoid mutations.

In our study, we used a variety of uridine and 5-substituted uridine analogs involving a primer extension by *Bst* DNA polymerase, and a good discrimination between A- and m^6^A-containing sequences (DNAs and RNAs) was achieved through a simple procedure. Moreover, the MD simulations demonstrate that the torsion angles for backbone conformations and puckered forms of the sugar ring vary quite significantly after methylation. This is accompanied by a monotonic widening of the minor groove. The model implies a loss of conformational freedom of binding interfaces between *Bst* DNA polymerase and the primer–template complex containing m^6^A, while this conformational flexibility is necessary for the processive movement of the enzyme.^22^ To some extent, m^6^A may cause processive *Bst* DNA polymerases to stall, thus leading to a block of the replication process.

Although m^6^A has been identified for a long time,^31^ there is an increasing demand to develop reliable and more efficient methods to unambiguously determine the exact position of this modification in RNA contexts,^32^ especially for analysing clinical specimens. To the best of our knowledge, the use of promoter methylation level as tumor biomarker has been intensively studied during the past decades.^33^ Since RNA is the downstream products of DNA during gene transcription, its methylation level can be potentially a more accurate tumor biomarker. To accomplish such a goal, we successfully used our method to determine the methylation status of specific sites in human rRNAs, using total RNAs extracted from cultured cells or clinical specimens. Evident differences in the RE values between the two groups are characterized and the results are statistically significant. The current study reveals that the human hepatic cancerous and paracancerous tissues are mainly methylated at position 1832 (18S RNA) and at position 4190 (28S RNA). Even more importantly, obviously lower levels of RNA methylation were characterized for hepatic cancerous tissues at positions 4189 and 4984 in the 28S subunit. At position 1781 in 18S subunit, a significantly higher level of methylation was observed in the cancerous pool, compared to the paracancerous control.

## Experimental section

### Detection of m^6^A in human rRNAs

For each reaction, 0.5 μg total RNA and each primer at 100 nM were used. This reaction was performed in 1× ThermoPol™ Reaction Buffer. A 20 μL sample was incubated in a water-bath at 45 °C for 1 h. The same protocol described in ESI† for RNA-directed DNA synthesis was used.

### Statistical analysis

Statistical analysis of rRNA methylation data was performed using the SPSS 19.0 software (SPSS Inc.). The methylation differences at each position between the two pools were tested with the Wilcoxon rank-sum test. Differences were considered to be significant for *P* < 0.05.

## Conclusions

Most importantly, we first reveal that significantly cancer-related changes in human rRNA methylation were present in patients with hepatocellular carcinoma. Although further evidence is needed to consolidate the connection between m^6^A and hepatic cancer, the methylation status of ‘A’ in target rRNA can potentially be treated as a novel tumor biomarker. Our findings can help advance understanding the function of this highly important modification in human disease.

## Supplementary Material

SC-007-C5SC02902C-s001
