# Supplementary material for: N 6-Methyladenine hinders RNA- and DNA-directed DNA synthesis: application in human rRNA methylation analysis of clinical specimens
Source: Chem Sci. 2015 Nov 17;7(2):1440–6. doi: 10.1039/c5sc02902c (PMC5975930; doi:10.1039/c5sc02902c)
Supplement: SC-007-C5SC02902C-s001 [file SC-007-C5SC02902C-s001.pdf]

Electronic Supplementary Material (ESI) for Chemical Science.
This journal is © The Royal Society of Chemistry 2015

Supporting Information

N[6]-methyladenine hinders RNA- and DNA- directed DNA synthesis: application in human rRNA methylation analysis of clinical specimens

Shaoru Wang[a,+], Jiaqi Wang[a,+], Xiaoe Zhang[a,+], Boshi Fu[a], Yanyan Song[a], Pei Ma[b], Kai Gu[a], Xin Zhou[b], Xiaolian Zhang[c], Tian Tian[a,*], Xiang Zhou[a,*]

Materials 2'-Deoxythymidine 5'-triphosphate (dTTP), 2'-deoxyuridine 5'-triphosphate (dUTP) and all the oligonucleotides were purchased from Takara Co. (Dalian, China). Bst DNA polymerase, Large Fragment was purchased from New England Biolabs, Inc. 5-hydroxymethyl-2'-deoxyuridine-5'-triphosphate (5-hmdUTP), 5-formyl-2'-deoxyuridine-5'-triphosphate (5-Formyl-dUTP), 5-fluoro-2'-deoxyuridine-5'-triphosphate (5-F-dUTP), 5-bromo-2'-deoxyuridine-5'-triphosphate (5-Br-dUTP) and 5-iodo-2'-deoxyuridine-5'-triphosphate (5-Iodo-dUTP) were purchased from Trilink Biotechnologies Inc. The chemical reagents were purchased from Sigma Inc. DMEM medium and Pen Strep antibiotic were purchased from Gibco, Life Technologies. Fetal Bovine Serum (FBS) was purchased from HyClone Inc. TRIzol was purchased from Invitrogen Inc. RNeasy Mini Kit was purchased from Qiagen Inc. Polyacrylamide gel electrophoresis products were finally scanned with Pharos FX Molecular imager (Bio-Rad, USA) operated in the fluorescence mode. The Discovery Series Quantity One 1-D Analysis Software Version 4.6.9 was used to determine the RE value. Background was manually subtracted.

DNA-directed DNA synthesis using *Bst* DNA polymerase This reaction was performed in 1 × ThermoPol™ Reaction Buffer (20 μL), which contained 20 mM Tris-HCl, 10 mM (NH4)2SO4, 10 mM KCl, 2 mM MgSO4 and 0.1 % Triton X-100 at pH 8.8 @ 25 °C, in the presence of 0.1 U Bst DNA polymerase, 200 nM primer, 40 μM dNTPs, 300 nM of template. After incubated at 45 °C for different times indicated on the gel, the reactions were quenched by adding 50 μL stop buffer (95% formamide, 25 mM EDTA pH 8.0), and the solutions were immediately heated at 90 °C for 10 min. After cooling down to 4 °C, the solutions were analyzed by denaturing 20% PAGE (19:1) in presence of 8 M urea.

Calculation study In MD simulation, the starting model structure was generated based on the reported crystal structure (PDB code 2BDP)[1]. 'A' to be investigated was modified using UCSF Chimera[2]. MD simulations were performed using PMEMD (Particle Mesh Ewald Molecular Dynamics) program in the Amber12 package[3] and the force field PARM99 parameter set [4] are employed for all simulations. The parameters for atom types in m^{6}A are taken from the GAFF [5] parameter set or developed by analogy to chemically similar atom types in the PARM99 and GAFF parameter sets. TIP3P model is used to perform the simulation of water molecules. Each system was solvated with approximately 29000 water molecules and sodium counterions are added for neutralization. For dealing with trajectories, UCSF Chimera

package was used for visualization and analysis [3b]. The solvated complex were equilibrated using a short minimization. And then 50 ps of heating and 50 ps of density equilibration with weak restraints on the complex were carried out. Finally, the molecular model was prepared in simulation environment after 500 ps of constant pressure equilibration at 300K (figure S14). All simulations were run with shake on hydrogen atoms, a 2 fs time step and langevin dynamics for temperature control. An ensemble of snapshots were obtained by recording the coordinates every 10 ps from 10 ns molecular simulation process after essential dynamics equilibration. The cpptraj modules of AMBER 12 was used to analyse the Trajectories. The effects of methyl group on 'm6A' in the template strand on backbone torsional angles and puckering were analysed. It has been shown that internal sugar ring bond angles are left flexible enough that sugar repuckering between C2'-endo and C3'-endo could be obtained [6]. That is, the energy barrier must be small enough for the sugar repuckering to occur. The conformation of a deoxyribose ring is closely correlated with the torsional angles (γ:O5'-C5'-C4'-C3' δ: C5'-C4'-C3'-O3') [7]. Hence, the changes in sugar pucker are perturbations to affect the replication process. We have investigated the torsion angle dynamics of m^6A and its perturbations in backbone conformation and sugar pucker, which are important for optimum replication [8].

Isolation of total RNA from HeLa cells HeLa cells and MCF-7 cells were obtained from CCTCC and grown in DMEM medium containing 10% Fetal Bovine Serum (FBS) and 1% Pen Strep at 37 °C, 5% CO_2 humidified atmosphere. A standard Trizol extraction was performed, and the aqueous phase was mixed with 1 volume of RNAse free ethanol, followed by the use of RNeasy Mini Kit. The following purification was performed according to the protocol by manufacturer. The RNA sample was quantitated on a NanoDrop 2000c (Thermo Scientific).

Isolation of total RNA from tissues This study was approved by the ethics committee of Zhongnan Hospital affiliated to Wuhan University. For research purposes, the hepatic cancerous or paracancerous tissues in patients with hepatocellular carcinoma were obtained from Zhongnan Hospital. All tissues were obtained with the approval of patients. All the patients were treated with surgery recently. Depending on the site and stage of the tumor, the cancerous tissue and some of the nearby one were removed. The nearby 2.0 cm of tissue from around the tumor site was removed and used as tested paracancerous control compared to cancerous tissue. For the assay, 20 mg of sample was used. Total RNA was extracted according to the manufacturer's protocol. The RNA sample was quantitated on a NanoDrop 2000c (Thermo Scientific).

Table S1. Sequences of oligomers used in our study

Oligomer	Sequence(from 5'to 3')
DNA-A	5'-CCC**A**CACCCTATAGTGAGTCGTA-3'
DNA-m^{6}A	5'-CCC-**m^{6}A**-CACCCTATAGTGAGTCGTA-3'
Primer 1	5'-FAM-ACTCACTATAGGGTG-3'
corA-non-A	5'-CTGG**A**GCACCGCCCGATCAGATCC-3'
corA-non-m6A	5'-CTGG-**m^{6}A**-GCACCGCCCGATCAGATCC-3'
Primer-non	5'-FAM-CTGATCGGGCGGTGC-3'
corA-temp-A	5'-CTCC**A**GGTTACGCGTCCCGGTATG-3'
corA-temp-m6A	5'-CTCC-**m^{6}A**-GGTTACGCGTCCCGGTATG-3'
Primer-temp	5'-FAM-CCGGGACGCGTAACC-3'
RNA-A	5'-AG**A**CUGCCACAUGCUGCAC-3'
RNA-m^{6}A	5'-AG-**m^{6}A**-CUGCCACAUGCUGCAC-3'
Primer 2	5'-FAM-GTGCAGCATGTGGCAG-3'
primer1781A	5'-FAM-CCTCTAGATAGTCAAGTTCGACCGTCTTCTCAGCGC-3'
primer1832mA	5'-FAM-TCCTTCCGCAGGTTCACCTACGGAAACCTTG-3'
primer4189	5'-FAM-AGCTCGCCTTAGGACACCTGCGT-3'
primer4190	5'-FAM-GAGCTCGCCTTAGGACACCTGCG-3'
primer4984A	5'-FAM-GCTGACTTTCAATAGATCGCAGCGAGGGAGC-3'

Table S2. The statistics of torsional angles for A during the MD simulation process

STATISTICS γ

AVERAGE: 48.8988 (14.5243 stddev)

INITIAL: 36.7754

FINAL: 51.2221

	g+	a+	t	a-	g-	c
%occupied	99.7					0.3
average	49.1					-10.4
stddev	14.1					

GAMMA canonical minor minor

O5'-C5'-C4'-C3', SNB range is 20-80 (g+)

STATISTICS δ

AVERAGE: 129.5491 (18.7795 stddev)

INITIAL: 142.2400

FINAL: 131.4229

	g+	a+	t	a-	g-	c
%occupied	5.3	84.7	10.0			
average	85.3	129.5	153.3			
stddev	3.6	15.0	3.2			

DELTA <------ canonical ------>

C5'-C4'-C3'-O3', SNB range is 70-180

DNA: ~128 with BI (a+), ~144 with BII (a+)

*** > 5% out of range population detected

STATISTICS χ

AVERAGE: -115.3560 (15.8615 stddev)

INITIAL: -91.4999

FINAL: -101.3806

	g+	a+	t	a-	g-	c
%occupied			3.0	94.7	2.3	

average			207.2	-114.9	-85.5	
stddev			2.4	14.1	4.0	

CHI <-------- anti -------> <--syn---

O4'-C1'-NX-CX, SNB range is 200-300

Table S3. The statistics of torsional angles for m^{6}A during the MD simulation process

---

STATISTICS γ

AVERAGE: 44.9508 (14.7540 stddev)

INITIAL: 39.6937

FINAL: -15.4824

	g+	a+	t	a-	g-	c
%occupied	98.0				0.7	1.3
average	46.5				-42.0	-22.0
stddev	10.3				2.1	5.7

GAMMA canonical minor minor

O5'-C5'-C4'-C3', SNB range is 20-80 (g+)

STATISTICS δ

AVERAGE: 134.6788 (11.1236 stddev)

INITIAL: 126.5888

FINAL: 123.0307

	g+	a+	t	a-	g-	c
%occupied		97.0	3.0			
average		134.1	153.2			
stddev		10.8	2.6			

DELTA <------ canonical ------>

C5'-C4'-C3'-O3', SNB range is 70-180

DNA: ~128 with BI (a+), ~144 with BII (a+)

STATISTICS χ

AVERAGE: -49.3208 (11.3174 stddev)

INITIAL: -56.7775

FINAL: -59.2001

	g+	a+	t	a-	g-	c
%occupied				0.3	96.7	3.0
average				-100.5	-49.9	-24.7
stddev					10.2	4.1

CHI <-------- anti -------> <--syn---

O4'-C1'-NX-CX, SNB range is 200-300

Table S4. Samples used in DNA methylation analysis

Name	Components (Final Conc.)
Sample 1	DNA-A (300 nM)
Sample 2	DNA-A (270 nM) and DNA-m^6A (30 nM)
Sample 3	DNA-A (240 nM) and DNA-m^6A (60 nM)
Sample 4	DNA-A (210 nM) and DNA-m^6A (90 nM)
Sample 5	DNA-A (180 nM) and DNA-m^6A (120 nM)
Sample 6	DNA-A (150 nM) and DNA-m^6A (150 nM)
Sample 7	DNA-A (120 nM) and DNA-m^6A (180 nM)
Sample 8	DNA-A (90 nM) and DNA-m^6A (210 nM)
Sample 9	DNA-A (60 nM) and DNA-m^6A (240 nM)
Sample 10	DNA-m^6A (300 nM)

Table S5. Samples used in RNA methylation analysis

Name	Components (Final Conc.)
Sample 1	RNA-A (400 nM)
Sample 2	RNA-A (360 nM) and RNA-m^6A (40 nM)
Sample 3	RNA-A (320 nM) and RNA-m^6A (80 nM)
Sample 4	RNA-A (280 nM) and RNA-m^6A (120 nM)
Sample 5	RNA-A (240 nM) and RNA-m^6A (160 nM)
Sample 6	RNA-A (200 nM) and RNA-m^6A (200 nM)
Sample 7	RNA-A (160 nM) and RNA-m^6A (240 nM)
Sample 8	RNA-A (120 nM) and RNA-m^6A (280 nM)
Sample 9	RNA-A (80 nM) and RNA-m^6A (320 nM)
Sample 10	RNA-m^6A (400 nM)

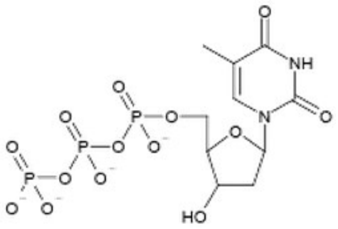

2'-Deoxythymidine 5'-triphosphate
(dTTP)

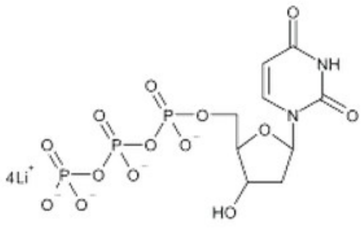

2'-deoxyuridine 5'-triphosphate
(dUTP)

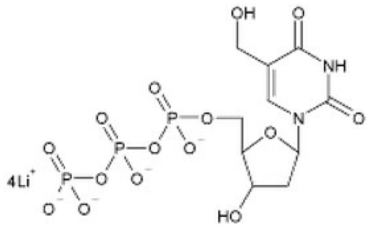

5-Hydroxymethyl-2’-deoxyuridine-5’-Triphosphate
(5-hmdUTP)

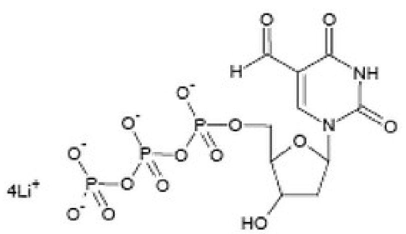

5-Formyl-2'-deoxyuridine-5'-Triphosphate
(5-Formyl-dUTP)

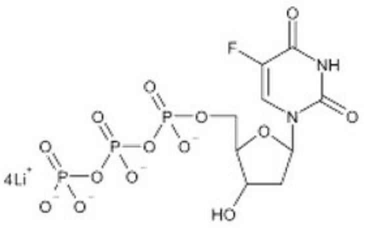

5-Fluoro-2'-deoxyuridine-5'-Triphosphate
(5-F-dUTP)

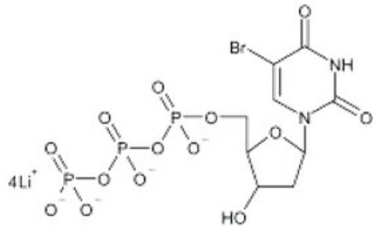

5-Bromo-2'-deoxyuridine-5'-Triphosphate
(5-Br-dUTP)

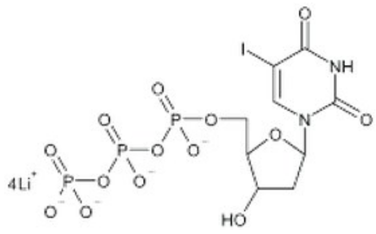

5-Iodo-2'-deoxyuridine-5'-Triphosphate
(5-Iodo-dUTP)

Figure S1. Structure illustrations of the studied 5'-triphosphates of a variety of 5-substituted 2'-deoxythymidine analogs.

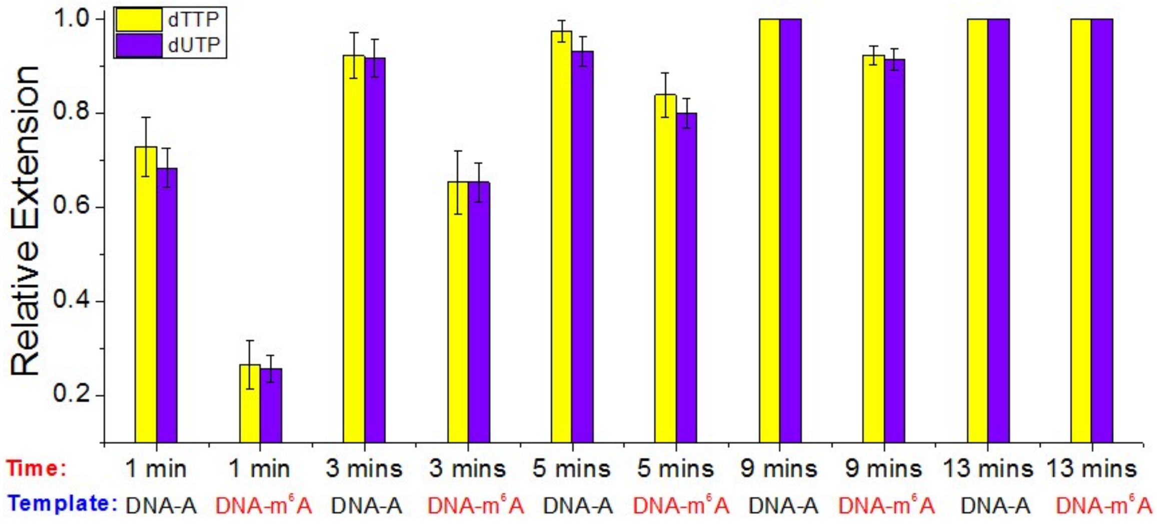

Figure S2. The analysis shows the difference of nucleotide (dTTP or dUTP) incorporation opposite a templating A or m^6A. The gel band intensities were converted into RE values, and all data are presented as the means ± SEM from three independent experiments. The error bars reflect the standard deviation.

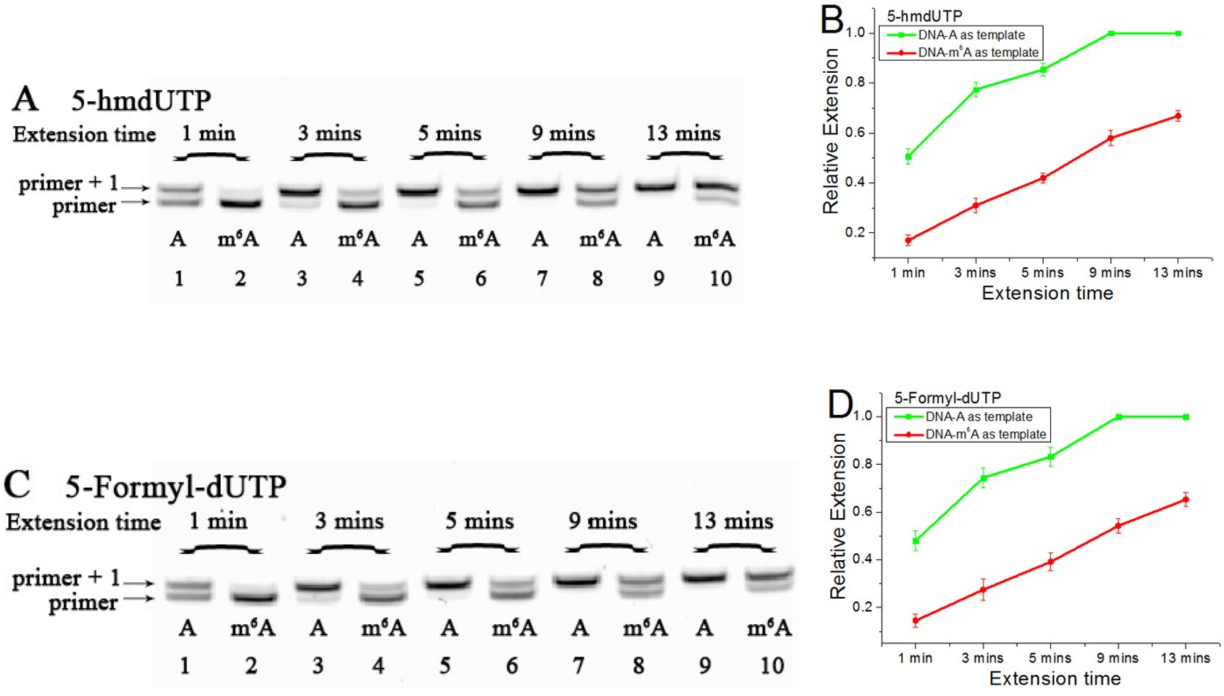

Figure S3. The analysis displays the difference of nucleotide incorporation opposite a templating A or m^{6}A. The extension condition is as following: 0.1 U *Bst* DNA polymerase at 45 ^{o}C for different times. Single nucleotide extended products ('primer + 1' band) gradually increase over elongation time. Lane 1, 3, 5, 7, 9, DNA-A was used as a template; lane 2, 4, 6, 8, 10, DNA-m^{6}A was used as a template. A) and C), representative gel image showing incorporation of 5-Hydroxymethyl-2'-deoxyuridine-5'-Triphosphate (5-hmdUTP) or 5-Formyl-2'-deoxyuridine-5'-Triphosphate (5-Formyl-dUTP); B) and D), all data are presented as the means ± SEM from three independent experiments, and the error bars reflect the standard deviation.

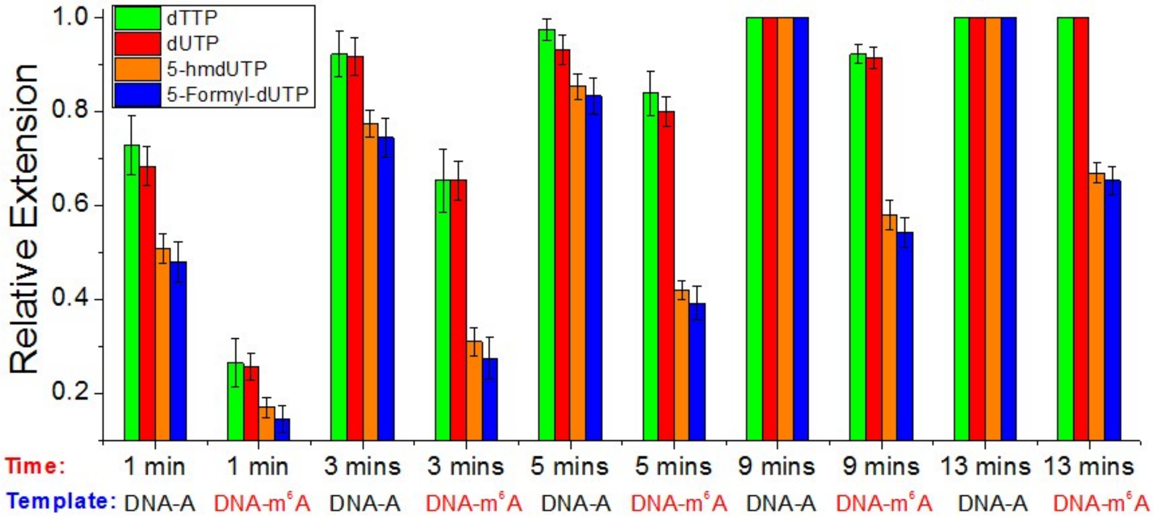

Figure S4. The analysis shows the difference of nucleotide (dTTP, dUTP, 5-hmdUTP or 5-Formyl-dUTP) incorporation opposite a templating A or m^{6}A. The extension condition is as following: 0.1 U *Bst* DNA polymerase at 45 ^{o}C for different time. The gel band intensities were converted into RE values, and all data are presented as the means ± SEM from three independent experiments. The error bars reflect the standard deviation.

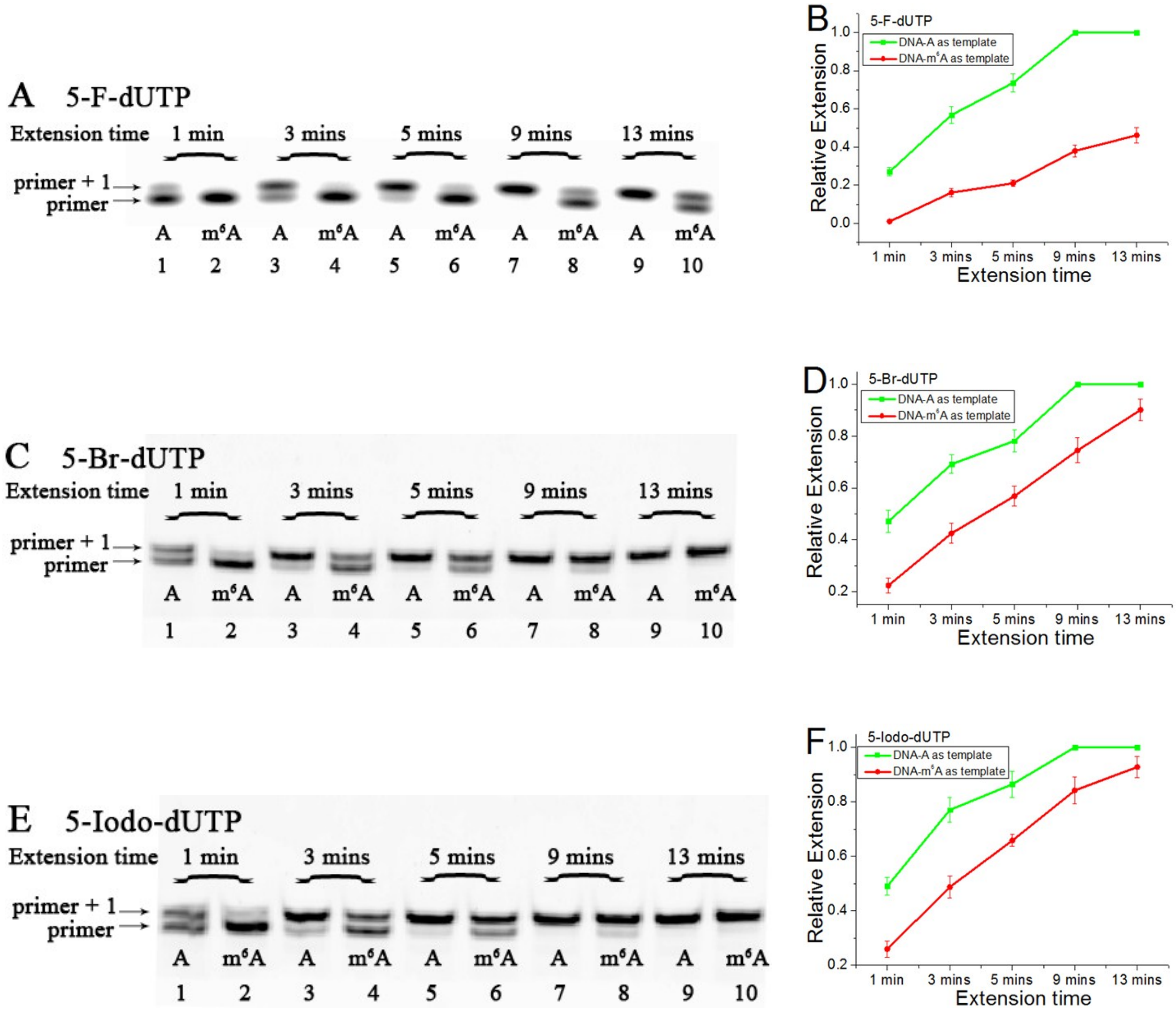

Figure S5. The analysis displays the difference of nucleotide incorporation opposite a templating A or m^{6}A. The extension condition is as following: 0.1 U *Bst* DNA polymerase at 45 ^{o}C for different times. Single nucleotide extended products (‘primer + 1’ band) gradually increase over elongation time. Lane 1, 3, 5, 7, 9, DNA-A was used as a template; lane 2, 4, 6, 8, 10, DNA-m^{6}A was used as a template. A), C) and E), representative gel image showing incorporation of 5-Fluoro-2'-deoxyuridine-5'-Triphosphate (5-F-dUTP), 5-Bromo-2'-deoxyuridine-5'-Triphosphate (5-Br-dUTP) or 5-Iodo-2'-deoxyuridine-5'-Triphosphate (5-Iodo-dUTP); B), D) and F), all data are presented as the means ± SEM from three independent experiments, and the error bars reflect the standard deviation.

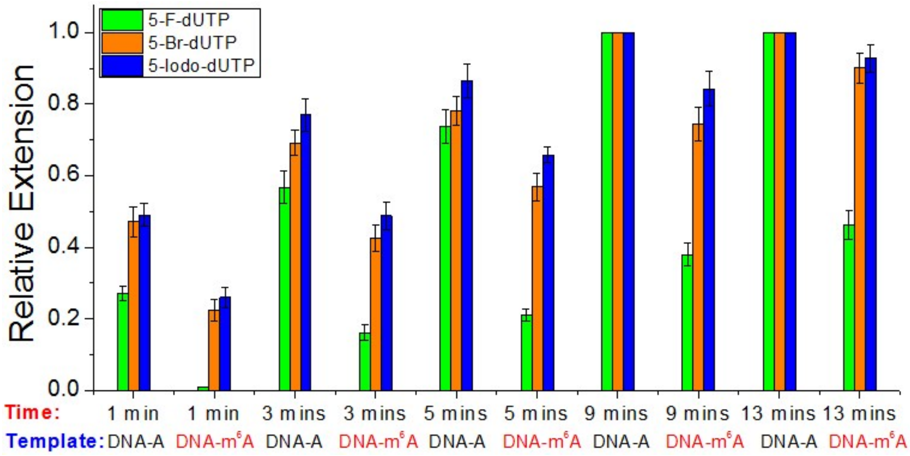

Figure S6. The analysis shows the difference of nucleotide incorporation opposite a templating A or m^{6}A. The gel band intensities were converted into RE values, and all data are presented as the means ± SEM from three independent experiments. The error bars reflect the standard deviation.

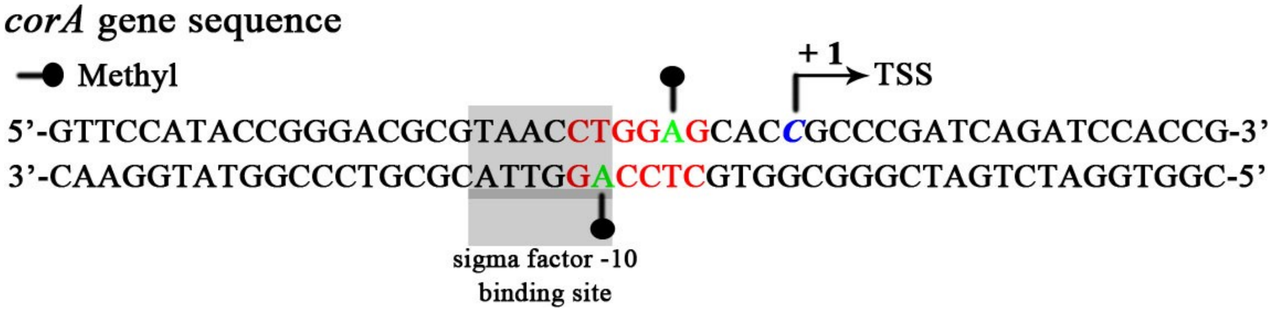

Figure S7. Illustration of partial genomic sequence around transcriptional start site (TSS) of corA gene in Mtb strain, H37Rv. The methylation region, TSSs and putative sigma factor -10 binding sites are shown as indicated.

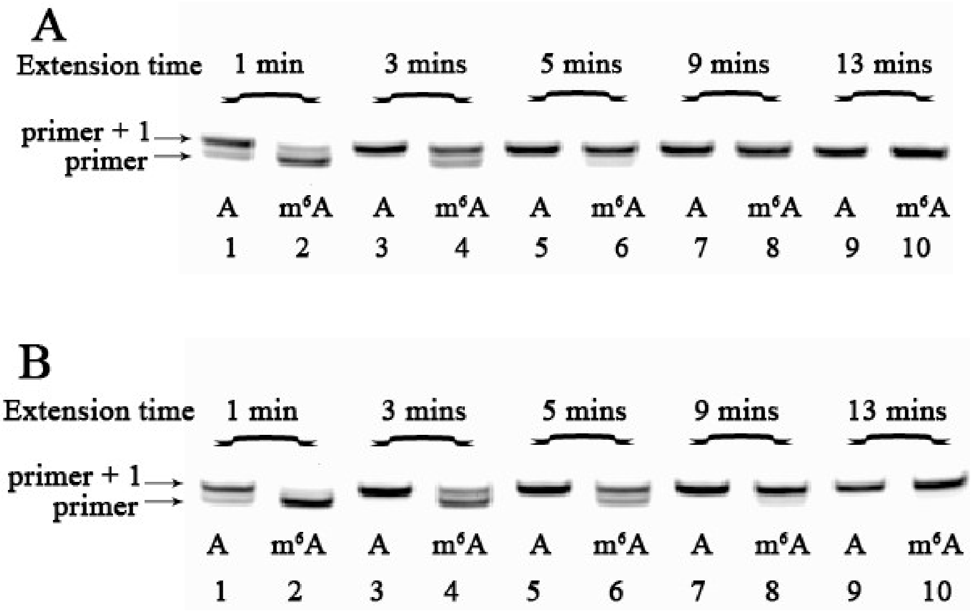

Figure S8. PAGE analysis displaying the difference in extension behaviour between *corA*-non-A and *corA*-non-m^{6}A. The extension condition is as following: 0.1 U *Bst* DNA polymerase at 45 ^{o}C for different times. Single nucleotide extended products ('primer + 1' band) gradually increase over elongation time. Extended DNAs were resolved on a 20% polyacrylamide denaturing gel using a 5'-FAM labelled primer. Lane 1, 3, 5, 7, 9, *corA*-non-A was used as a template; lane 2, 4, 6, 8, 10, *corA*-non-m^{6}A was used as a template. A), dTTP was incorporated into the growing DNA chain; B), 2'-deoxyuridine-5'-Triphosphate (dUTP) was incorporated into the growing DNA chain.

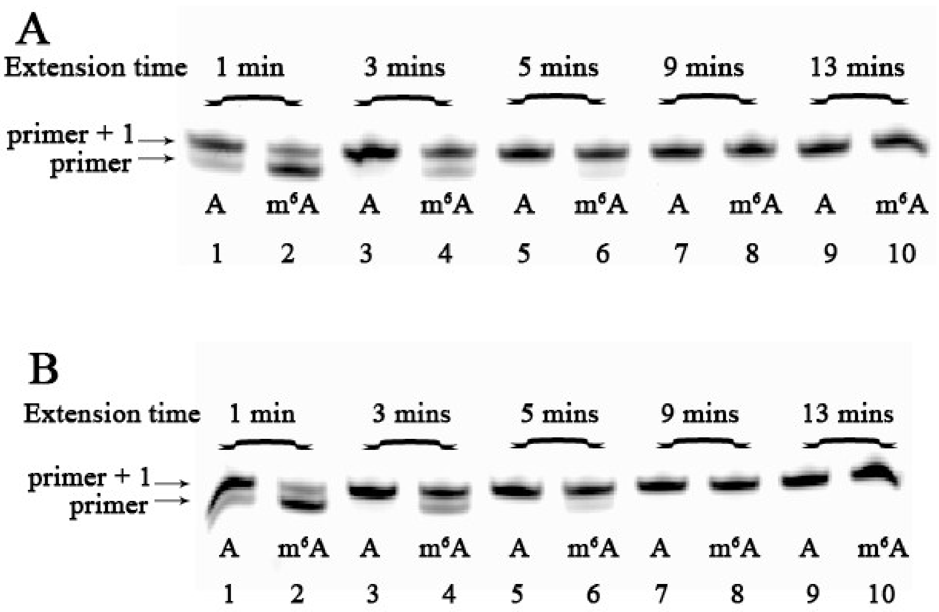

Figure S9. PAGE analysis displaying the difference in extension behaviour between *corA*-temp-A and *corA*-temp-m^{6}A. The extension condition is as following: 0.1 U *Bst* DNA polymerase at 45 ^{o}C for different times. Single nucleotide extended products ('primer + 1' band) gradually increase over elongation time. Extended DNAs were resolved on a 20% polyacrylamide denaturing gel using a 5'-FAM labelled primer. Lane 1, 3, 5, 7, 9, *corA*-temp-A was used as a template; lane 2, 4, 6, 8, 10, *corA*-temp-m^{6}A was used as a template. A), dTTP was incorporated into the growing DNA chain; B), 2'-deoxyuridine-5'-Triphosphate (dUTP) was incorporated into the growing DNA chain.

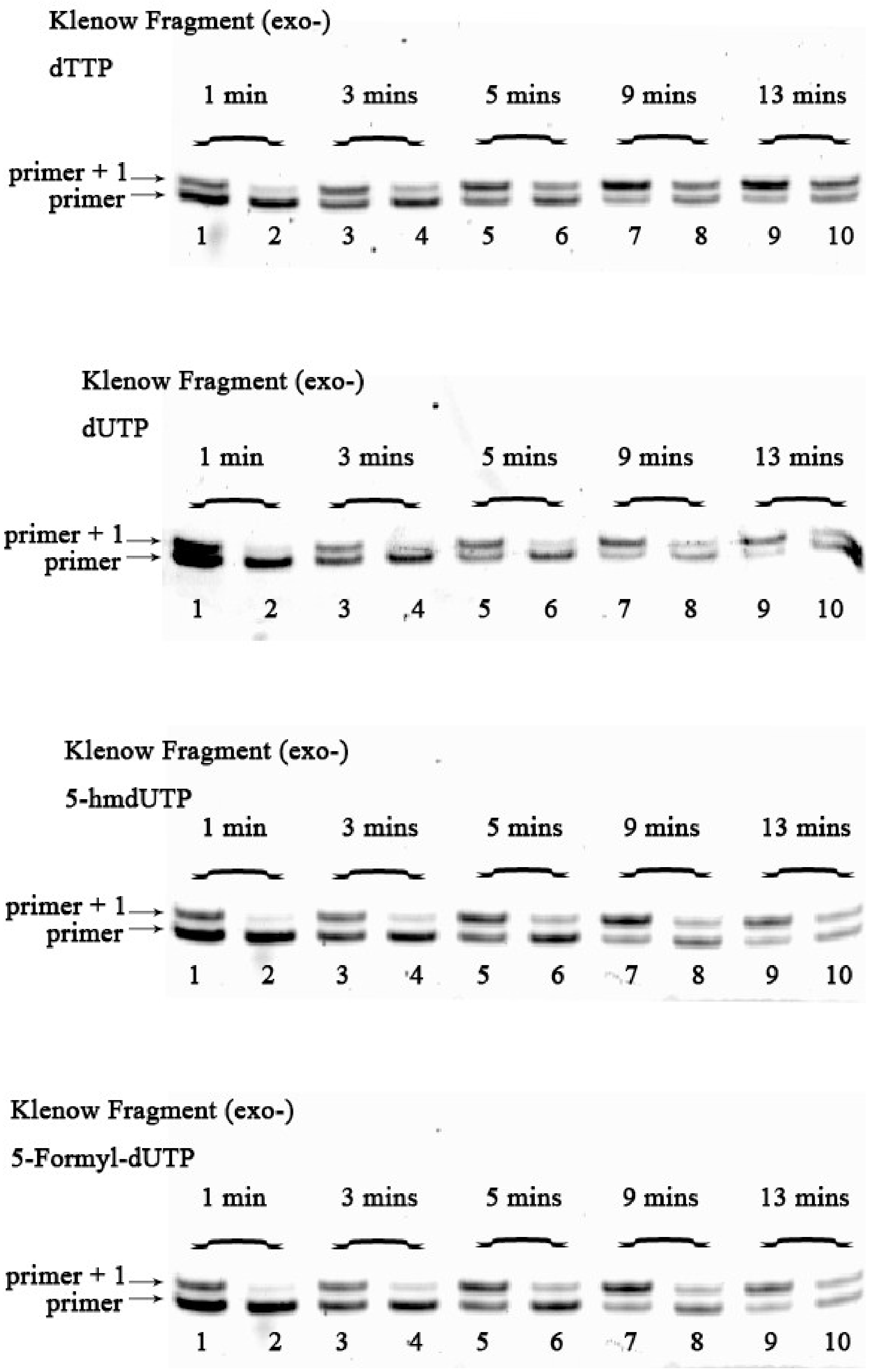

Figure S10. The analysis displays the difference of nucleotide incorporation opposite a templating A or m^6A. Lane 1, 3, 5, 7, 9, DNA-A was used as a template; lane 2, 4, 6, 8, 10, DNA-m^6A was used as a template. The extension condition is as following: 0.1 U Klenow Fragment (exo-) at 37 ^oC for different durations. Single nucleotide extended products ('primer + 1' band) gradually increase versus elongation time. Extended DNAs were resolved on a 20% polyacrylamide denaturing gel.

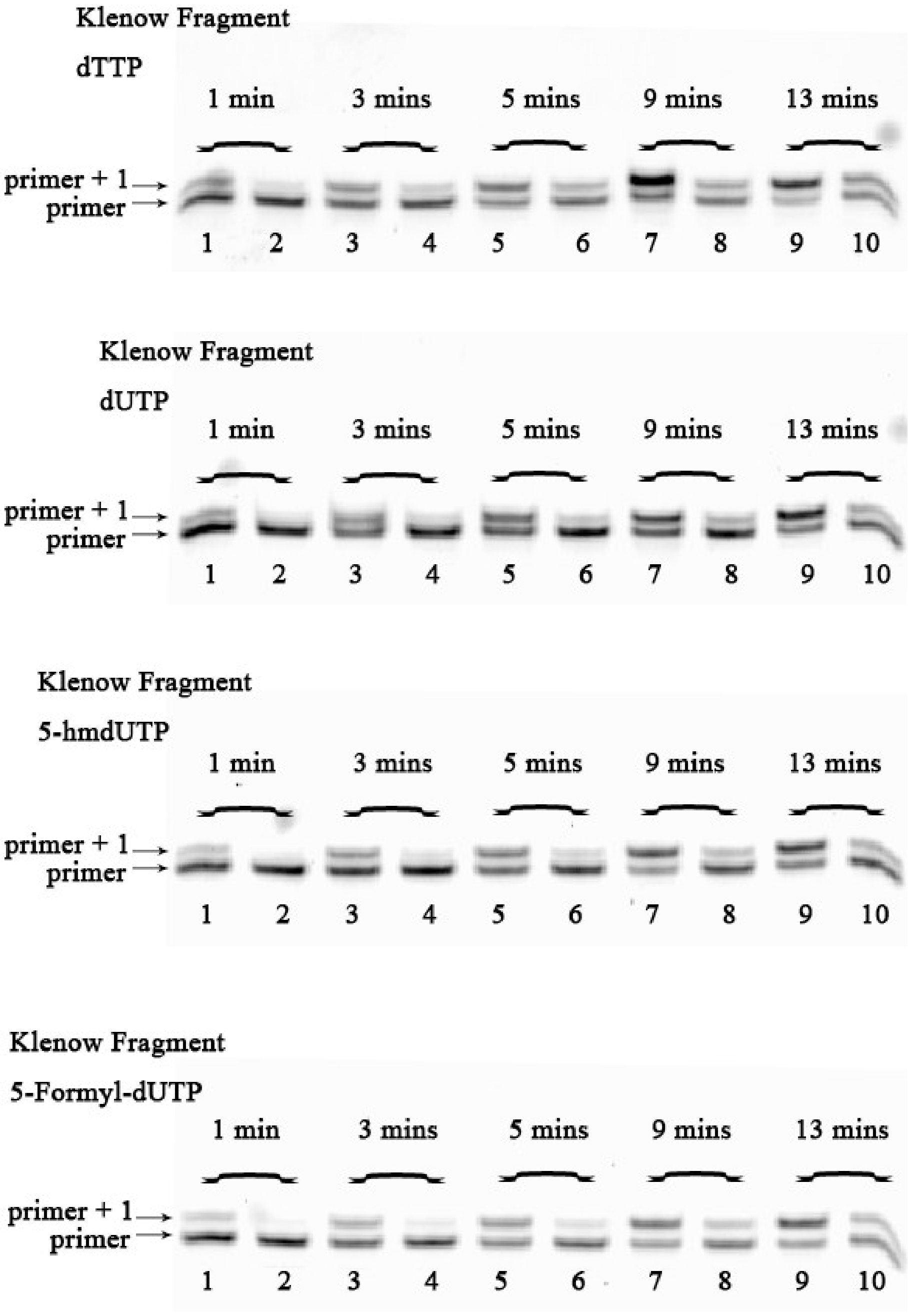

Figure S11. The analysis displays the difference of nucleotide incorporation opposite a templating A or m^{6}A. Lane 1, 3, 5, 7, 9, DNA-A was used as a template; lane 2, 4, 6, 8, 10, DNA-m^{6}A was used as a template. The extension condition is as following: 0.1 U Klenow Fragment at 37 ^{o}C for different durations. Single nucleotide extended products (‘primer + 1’ band) gradually increase versus elongation time. Extended DNAs were resolved on a 20% polyacrylamide denaturing gel.

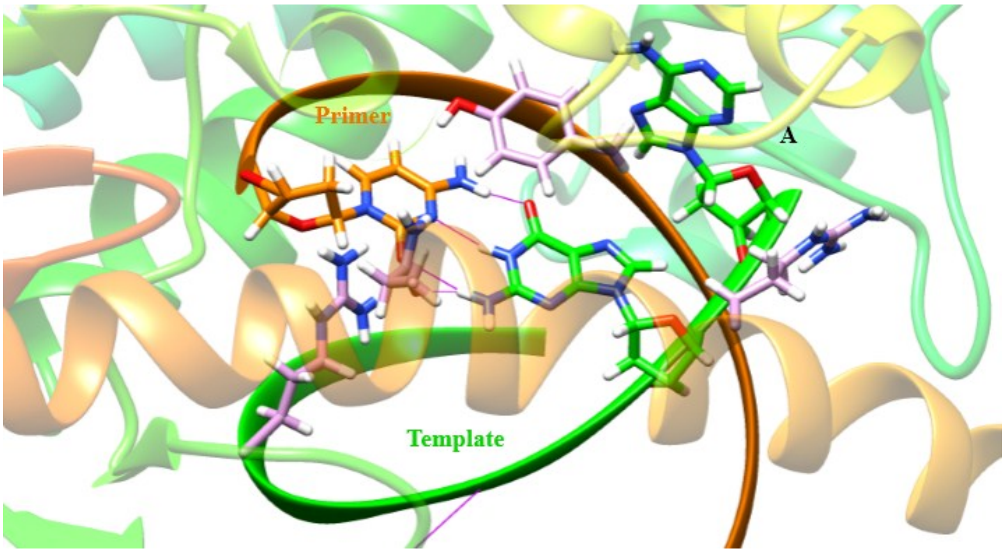

Figure S12. A structure model of the primer/template complex (containing A) in the *Bst* DNA polymerase active site place. The starting structure was generated by using the deposited crystal structure (PDB code 2BDP). The structure is superposed with the template–primer (green/orange) duplex. Key residues in the MGR region of enzyme (pink) were displayed, including Tyr 714 (766), Arg 615 (668) and Gln 797 (849). The protein-DNA interface in the MGR region is stabilized by the hydrogen bonds (H-bonds, violet) and the stacking interaction between the template base and the corresponding residue [Tyr 714 (766)].

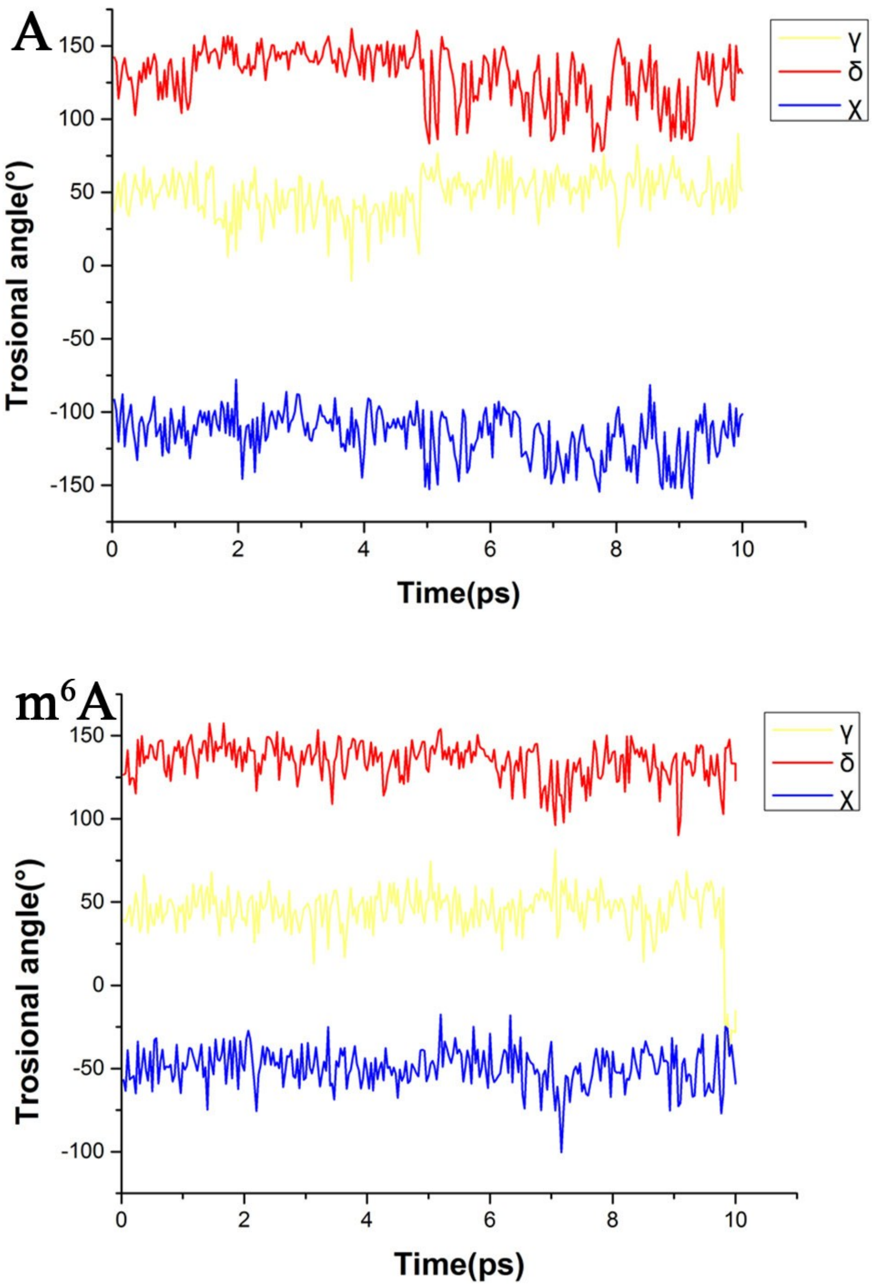

Figure S13. The plots of the distribution of backbone torsional angles during the MD simulations.

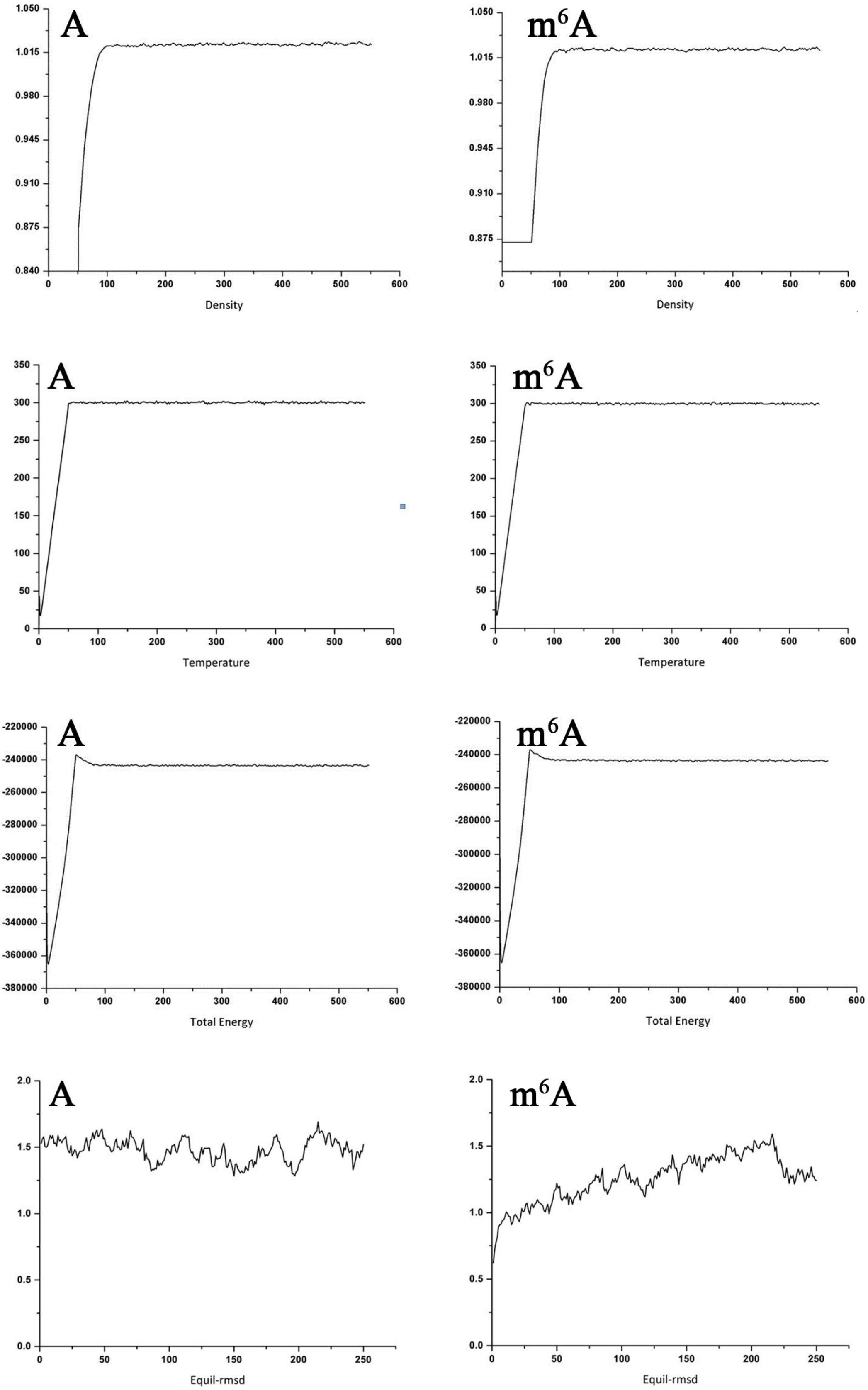

Figure S14. The plots of density, temperature and total energy were all clearly converged at the end of the equilibration period. The time evolution of root mean square deviation (RMSD) of the backbone atoms about their average position was displayed. During the simulation, the complex held together and the stable trajectories were obtained.

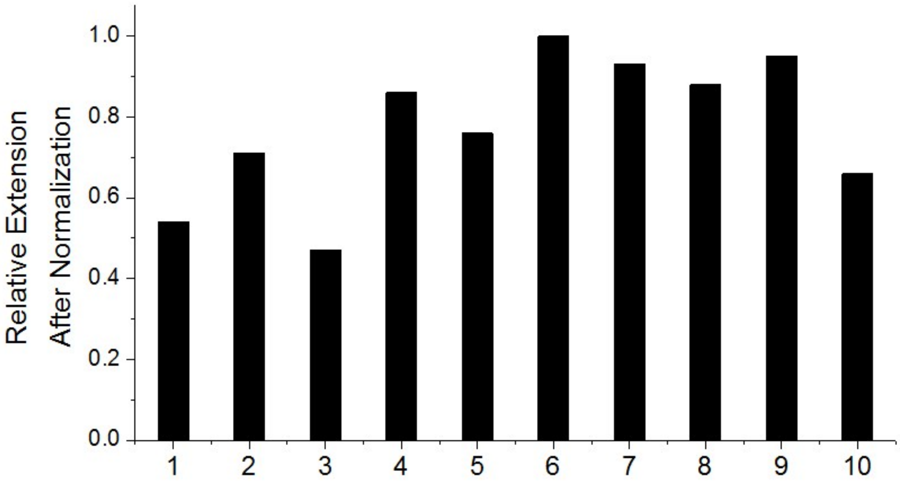

Figure S15. Extension behaviour of 5-hmdUTP between DNA-A and DNA-m^{6}A, by alteration of the thermal extension time, enzyme concentration, as well as the extension temperature. Extended DNAs were resolved on a 20% polyacrylamide denaturing gel using a 5'-FAM labelled primer. For all experiments, the gel band intensities were converted into relative extension values. Relative extension was normalized (relative to the highest as 100%). *Bst* DNA polymerase was used in the assay. 1, 12 mins, 0.05 U and 43 ^{o}C; 2, 12 mins, 0.05 U and 47 ^{o}C; 3, 12 mins, 0.06 U and 43 ^{o}C; 4, 12 mins, 0.06 U and 47 ^{o}C; 5, 13 mins, 0.05 U and 43 ^{o}C; 6, 13 mins, 0.05 U and 45 ^{o}C; 7, 13 mins, 0.05 U and 47 ^{o}C; 8, 13 mins, 0.04 U and 45 ^{o}C; 9, 13 mins, 0.06 U and 44 ^{o}C; 10, 14 mins, 0.04 U and 45 ^{o}C.

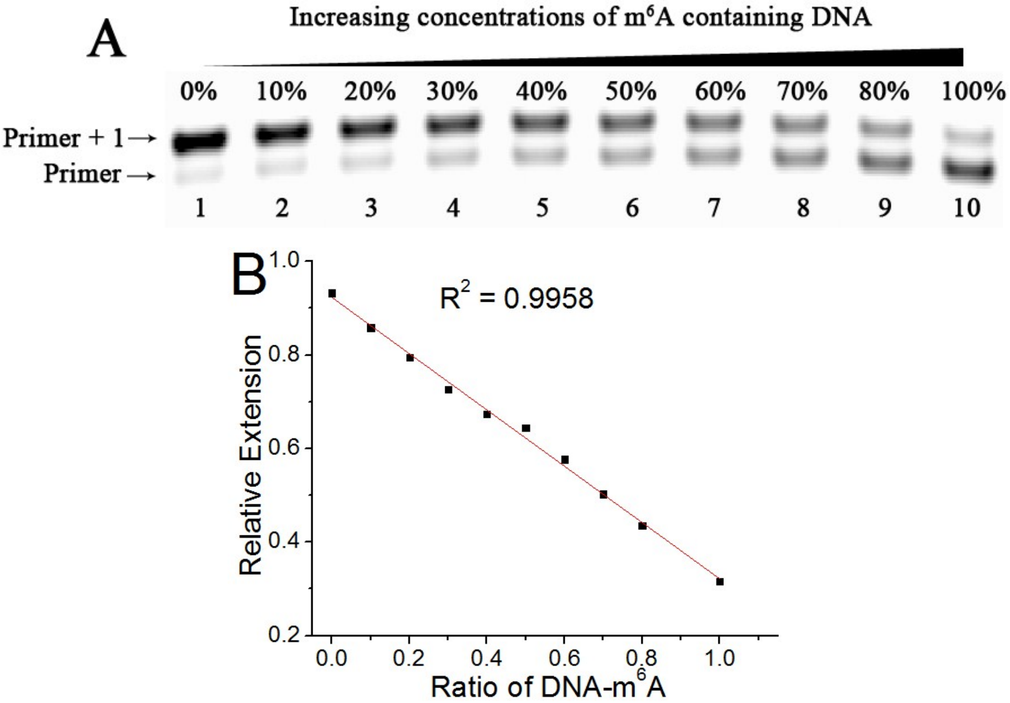

Figure S16. The values of RE correlate inversely linearly with the fraction of DNA-m^{6}A. 5-hmdUTP is used in the analysis. The extension condition is as following: 0.05 U *Bst* DNA polymerase at 45 ^{o}C for 13 mins. A). Lane 1 - lane 10, defined fractions of DNA-m^{6}A were used (0, 10%, 20%, 30%, 40%, 50%, 60%, 70%, 80% and 100%). B). Standard calibration curve: RE versus different ratios of DNA-m^{6}A (R^2 = 0.9958).

A).

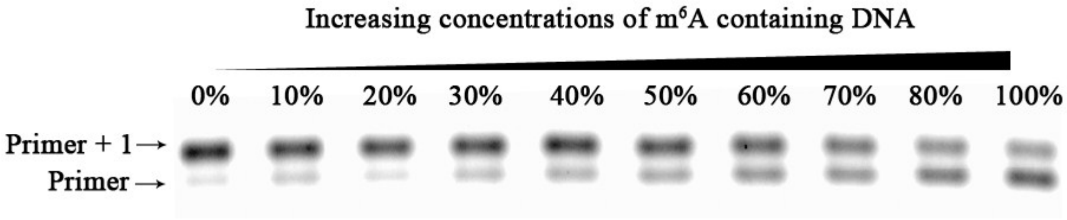

B).

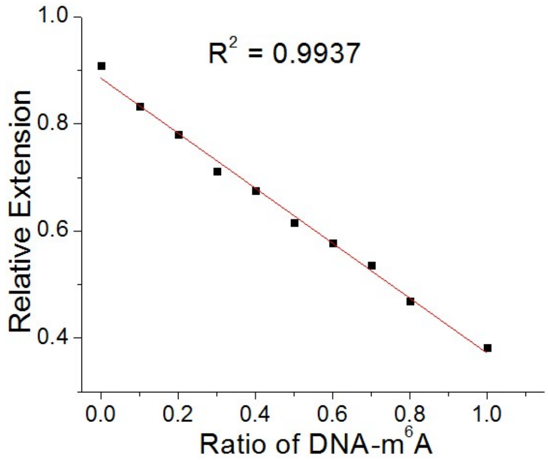

Figure S17. The values of RE correlate inversely linearly with the fraction of DNA-m6A. 5-Formyl-dUTP is used in the analysis. The extension condition is as following: 0.05 U *Bst* DNA polymerase at 45 ^{o}C for 13 mins. A). Lane 1 - lane 10, defined fractions of DNA-m6A were used (0, 10%, 20%, 30%, 40%, 50%, 60%, 70%, 80% and 100%). B). Standard calibration curve: RE versus different ratios of DNA-m6A (R^2 = 0.9937).

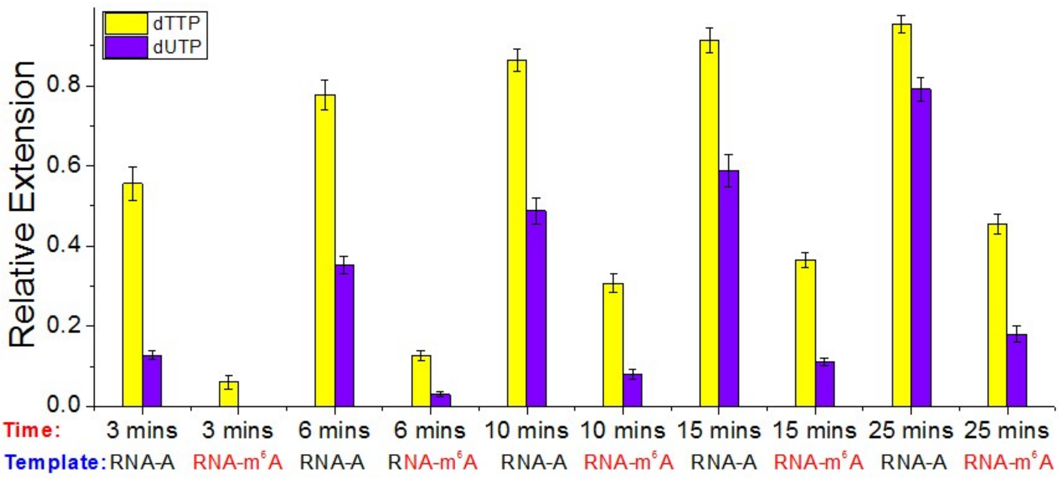

Figure S18. The analysis shows the difference of nucleotide (dTTP or dUTP) incorporation opposite a templating A or m^{6}A. The extension condition is as following: 0.1 U *Bst* DNA polymerase at 45 ^{o}C for different time. The gel band intensities were converted into RE values, and all data are presented as the means ± SEM from three independent experiments. The error bars reflect the standard deviation.

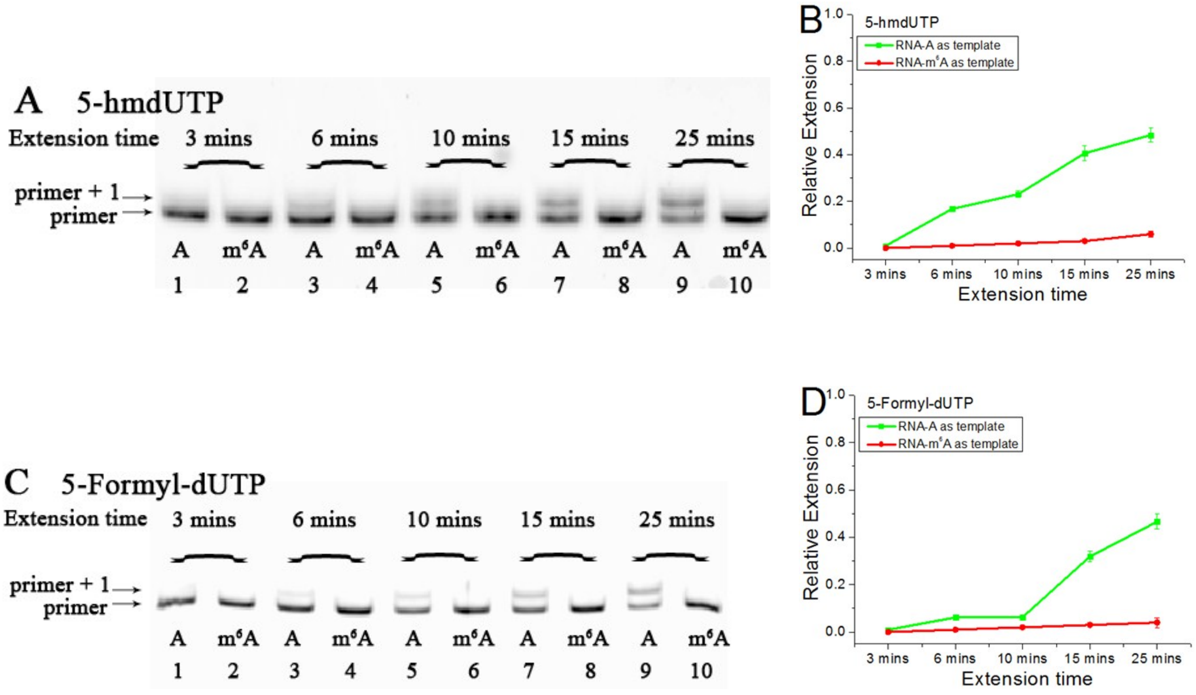

Figure S19. The analysis displays the difference of nucleotide incorporation opposite a templating A or m^{6}A. The extension condition is as following: 0.1 U *Bst* DNA polymerase at 45 ^{o}C for different time. Single nucleotide extended products (‘primer + 1’ band) gradually increase over elongation time. Lane 1, 3, 5, 7, 9, RNA-A was used as a template; lane 2, 4, 6, 8, 10, RNA-m^{6}A was used as a template. A) and C), representative gel image showing incorporation of 5-Hydroxymethyl-2’-deoxyuridine-5’-Triphosphate (5-hmdUTP) or 5-Formyl-2'-deoxyuridine-5'-Triphosphate (5-Formyl-dUTP); B) and D), all data are presented as the means ± SEM from three independent experiments, and the error bars reflect the standard deviation.

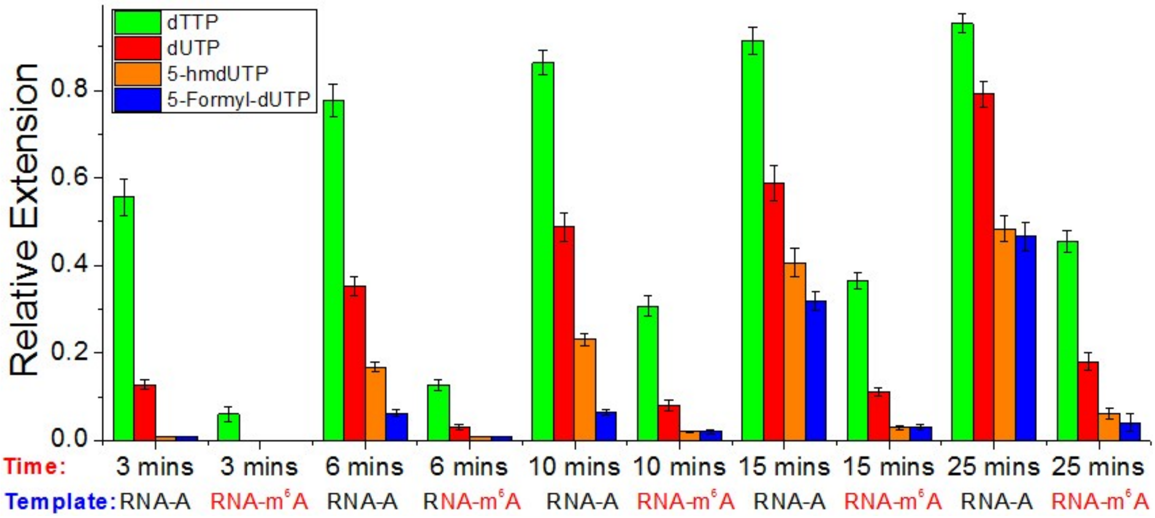

Figure S20. The analysis shows the difference of nucleotide (dTTP, dUTP, 5-hmdUTP or 5-Formyl-dUTP) incorporation opposite a templating A or m^{6}A. The extension condition is as following: 0.1 U *Bst* DNA polymerase at 45 ^{o}C for different time. The gel band intensities were converted into RE values, and all data are presented as the means ± SEM from three independent experiments. The error bars reflect the standard deviation.

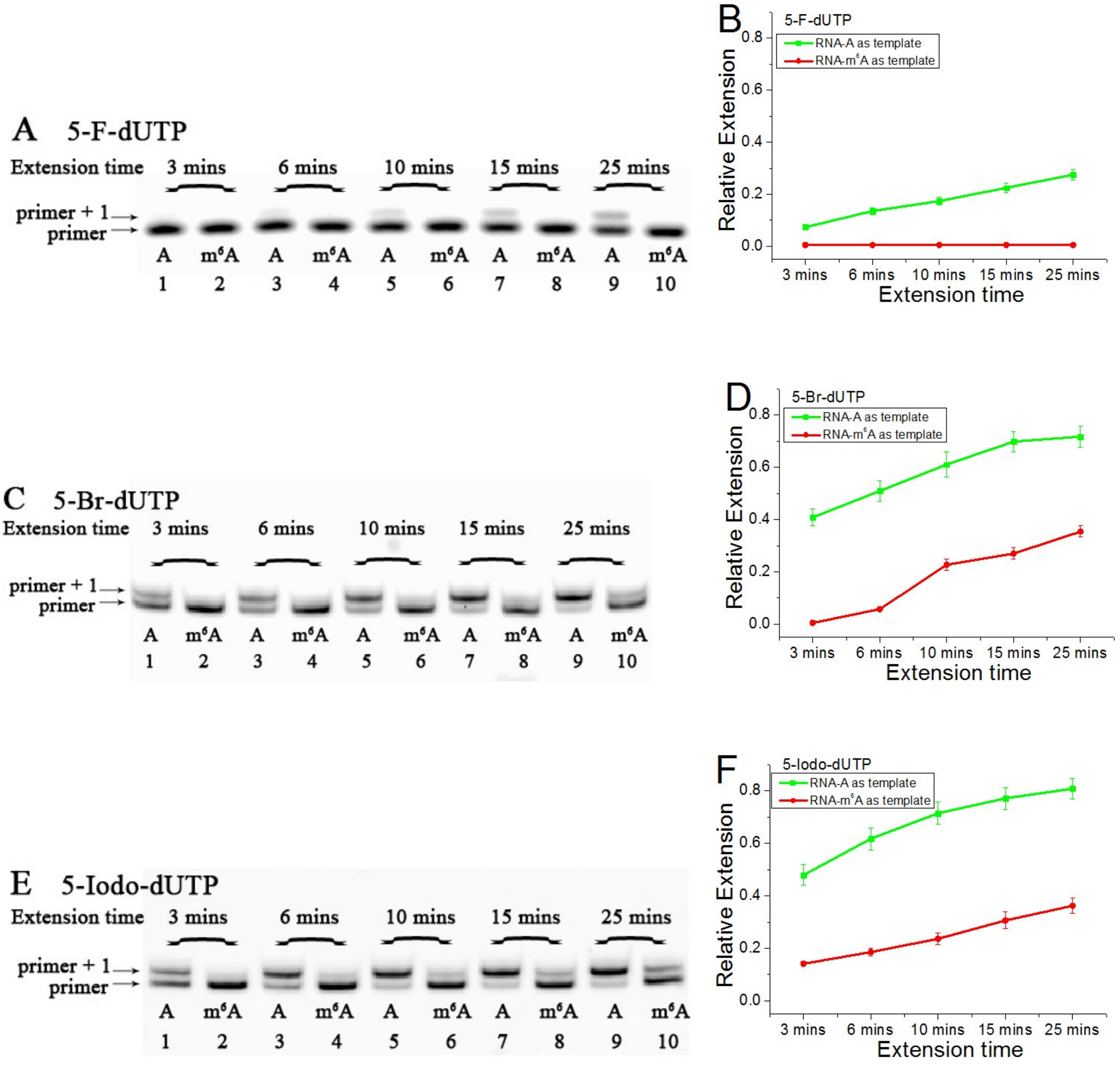

Figure S21. The analysis displays the difference of nucleotide incorporation opposite a templating A or m^{6}A. The extension condition is as following: 0.1 U *Bst* DNA polymerase at 45 ^{o}C for different time. Single nucleotide extended products ('primer + 1' band) gradually increase over elongation time. Lane 1, 3, 5, 7, 9, RNA-A was used as a template; lane 2, 4, 6, 8, 10, RNA-m^{6}A was used as a template. A), C) and E), representative gel image showing incorporation of 5-Fluoro-2'-deoxyuridine-5'-Triphosphate (5-F-dUTP), 5-Bromo-2'-deoxyuridine-5'-Triphosphate (5-Br-dUTP) or 5-Iodo-2'-deoxyuridine-5'-Triphosphate (5-Iodo-dUTP); B), D) and F), all data are presented as the means ± SEM from three independent experiments, and the error bars reflect the standard deviation.

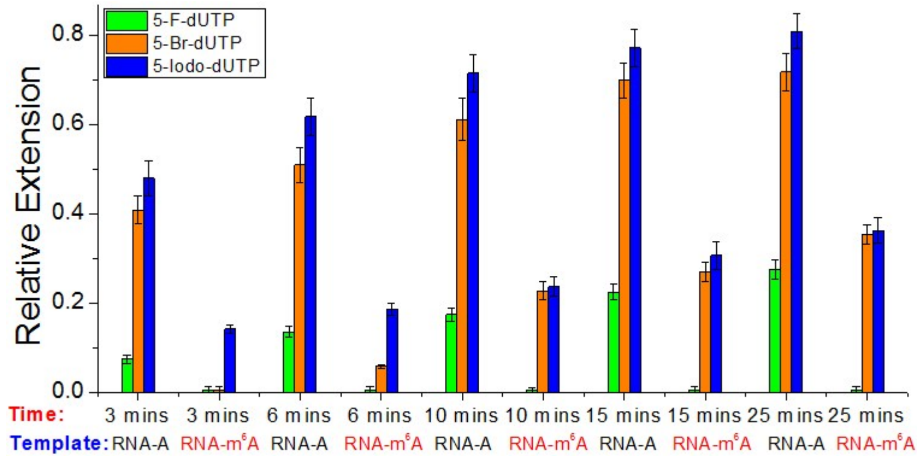

Figure S22. The analysis shows the difference of nucleotide incorporation opposite a templating A or m6A. The gel band intensities were converted into RE values, and all data are presented as the means ± SEM from three independent experiments. The error bars reflect the standard deviation.

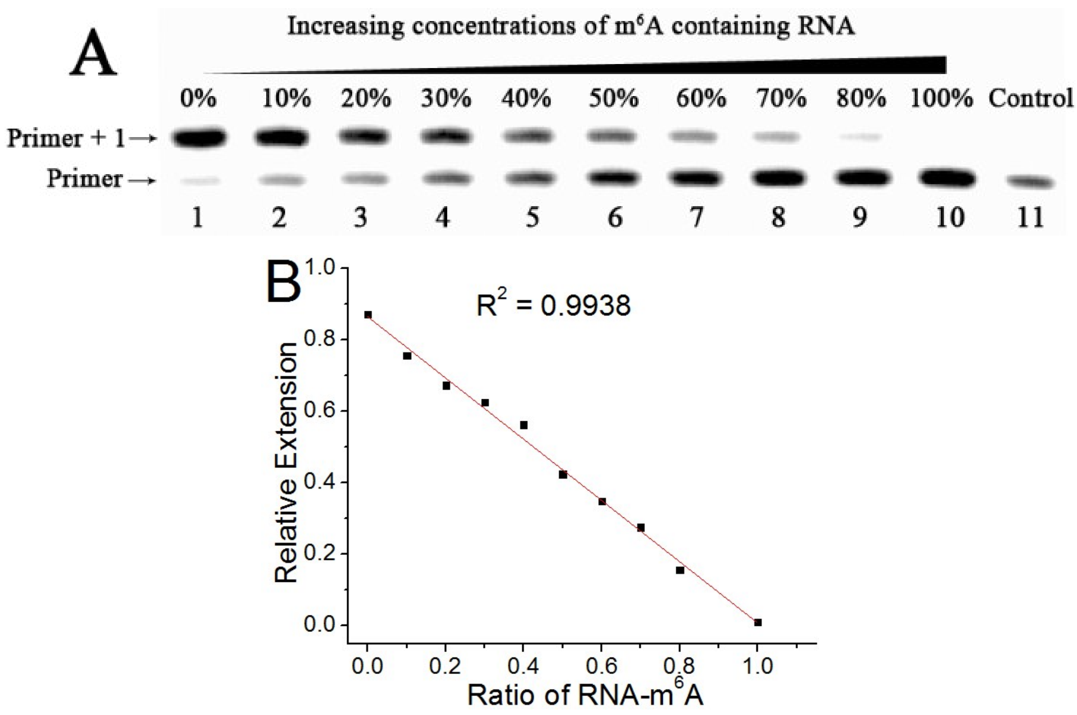

Figure S23. The values of RE correlate inversely linearly with the fraction of RNA-m^{6}A. 5-Iodo-dUTP is incorporated for analysis. The extension condition is as following: 0.06 U *Bst* DNA polymerase at 45 ^{o}C for 12 mins. A). Lane 1 - lane 10, defined fractions of RNA-m^{6}A were used (0, 10%, 20%, 30%, 40%, 50%, 60%, 70%, 80% and 100%). Lane 11, primer 2 was used as a marker. B). Standard calibration curve: RE versus different ratios of RNA-m^{6}A (R^2 = 0.9938).

A).

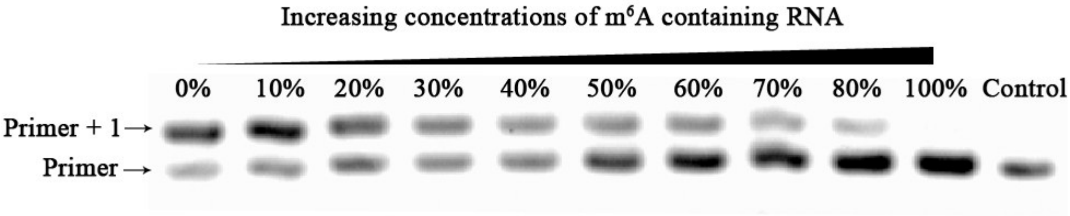

B).

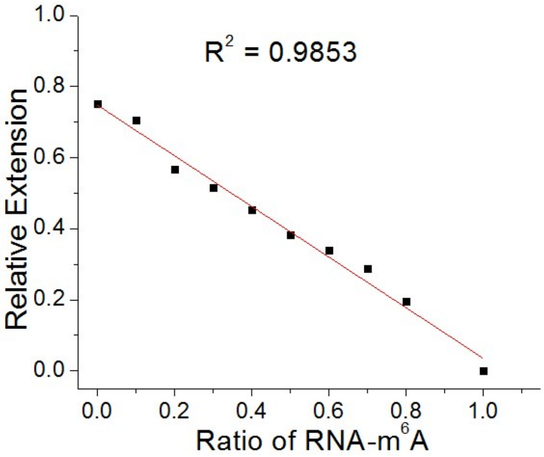

Figure S24. The values of RE correlate inversely linearly with the fraction of RNA-m^{6}A. dTTP is incorporated for analysis. The extension condition is as following: 0.06 U *Bst* DNA polymerase at 45 ^{o}C for 12 mins. A). Lane 1 - lane 10, defined fractions of RNA-m^{6}A were used (0, 10%, 20%, 30%, 40%, 50%, 60%, 70%, 80% and 100%). Lane 11, primer 2 was used as a marker. B). Standard calibration curve: RE versus different ratios of RNA-m^{6}A (R^2 = 0.9853).

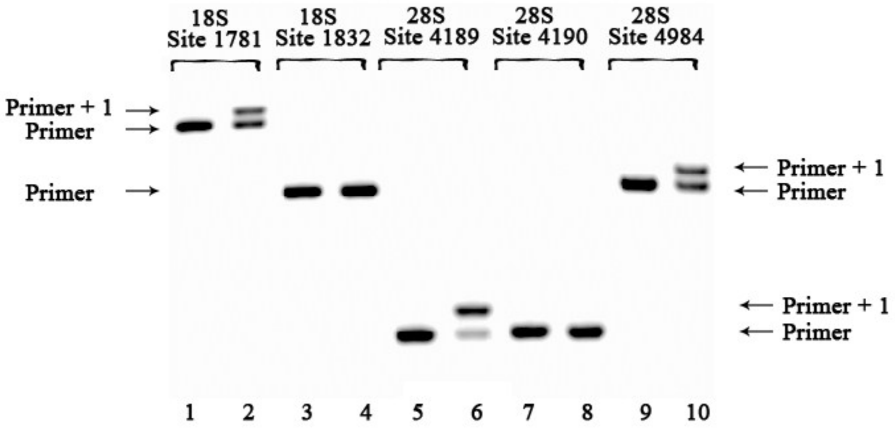

Figure S25. Probing methylated and non-methylated A sites in 18S subunit and 28S subunit of human rRNA. Cultured HeLa tumor cells were used for analysis. Lanes 1, 3, 5, 7 and 9, control samples without addition of *Bst* DNA polymerase; lanes 2, 4, 6, 8 and 10, 0.1 U *Bst* DNA polymerase was used.

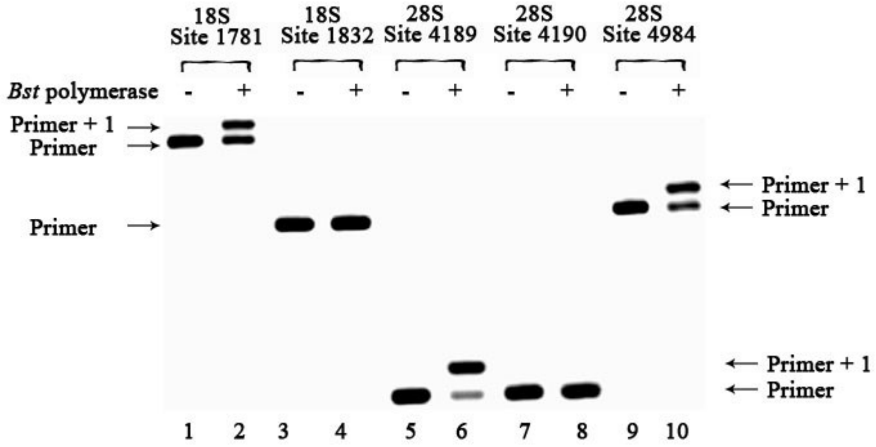

Figure S26. Probing methylated and non-methylated A sites in 18S subunit and 28S subunit of human rRNA. Cultured MCF-7 tumor cells were used for analysis. The extension condition is as following: heating at 45 ^{o}C for 1 hour. The analysis shows the difference in extension behaviour between the studied sites. Extended DNAs were resolved on a 20% polyacrylamide denaturing gel. Lanes 1, 3, 5, 7 and 9, control samples without addition of *Bst* DNA polymerase; lanes 2, 4, 6, 8 and 10, 0.1 U *Bst* DNA polymerase was used.

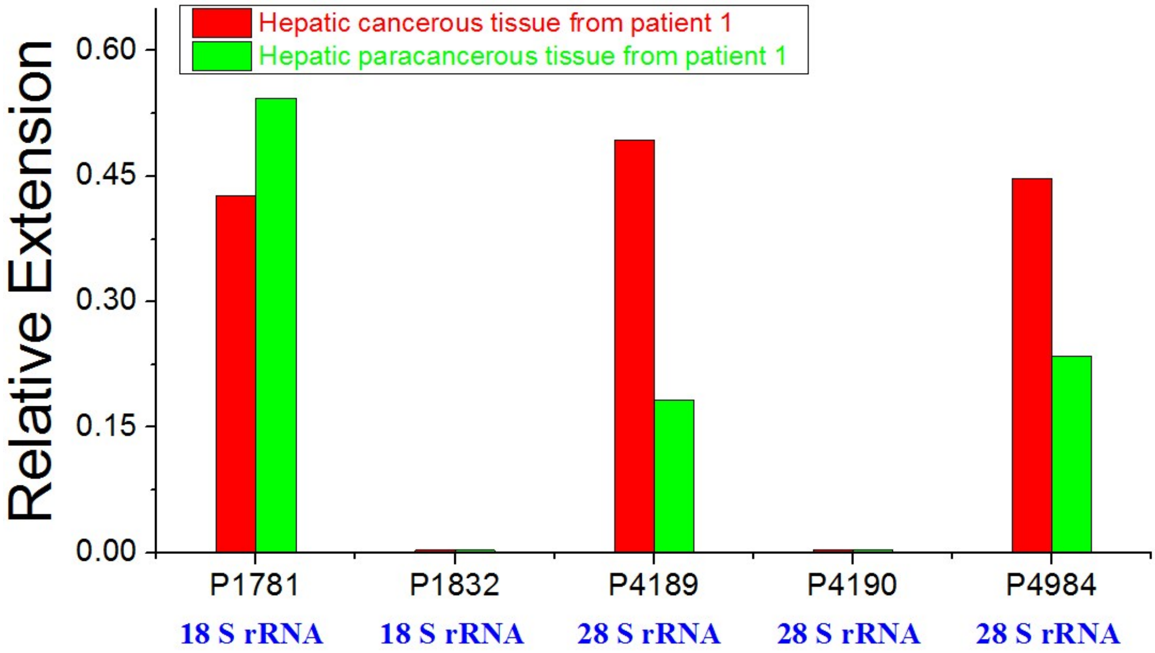

Figure S27. Methylation analysis of 18S and 28S rRNA for hepatic cancerous or paracancerous tissue from a same patient with hepatocellular carcinoma. dTTP is incorporated for analysis. For all experiments, the gel band intensities were converted into RE values. The analysis displays the difference in extension behaviour between hepatic cancerous and paracancerous tissues of the same patient.

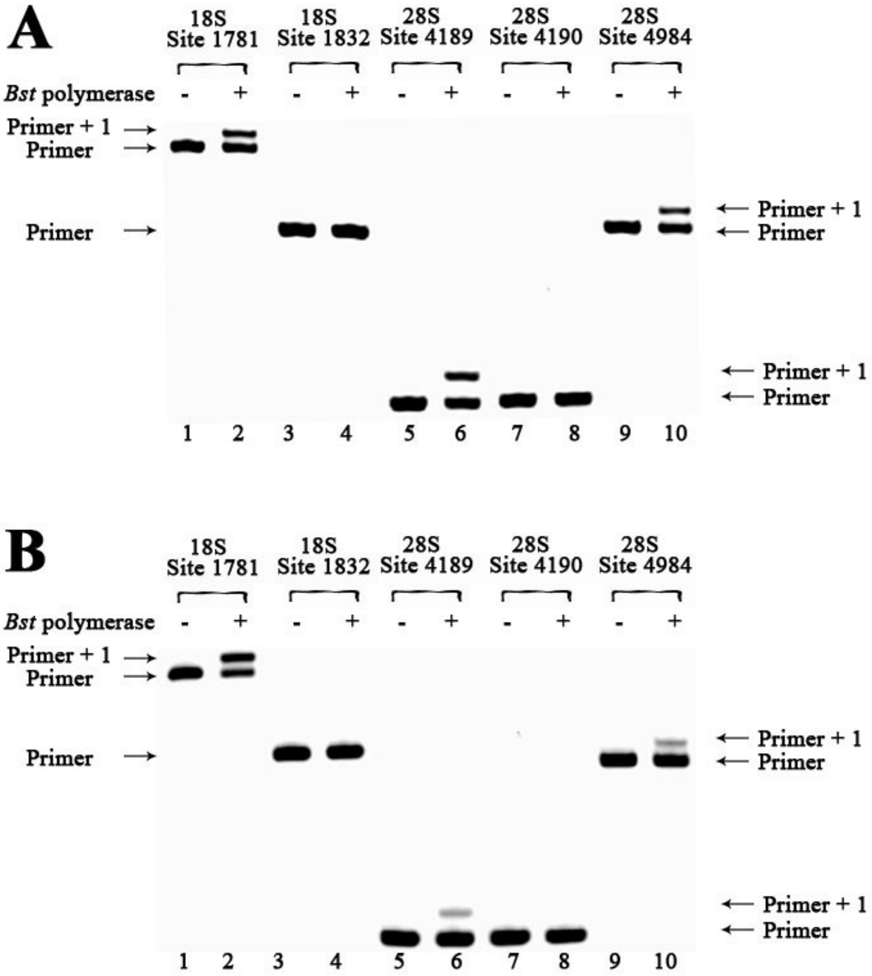

Figure S28. 18S and 28S rRNA methylation for clinical specimens was examined. 5'-FAM labelled primers were used the same as previous studies. The extension condition is as following: heating at 45 ^{o}C for 1 hour. The analysis shows the difference in extension behaviour between the studied sites. Extended DNAs were resolved on a 20% polyacrylamide denaturing gel. Lanes 1, 3, 5, 7 and 9, control samples without addition of *Bst* DNA polymerase; lanes 2, 4, 6, 8 and 10, 0.1 U *Bst* DNA polymerase was used. A) analysis for hepatic cancerous tissues of patient 2 with hepatocellular carcinoma; B) analysis for hepatic paracancerous tissues of the same patient with hepatocellular carcinoma.

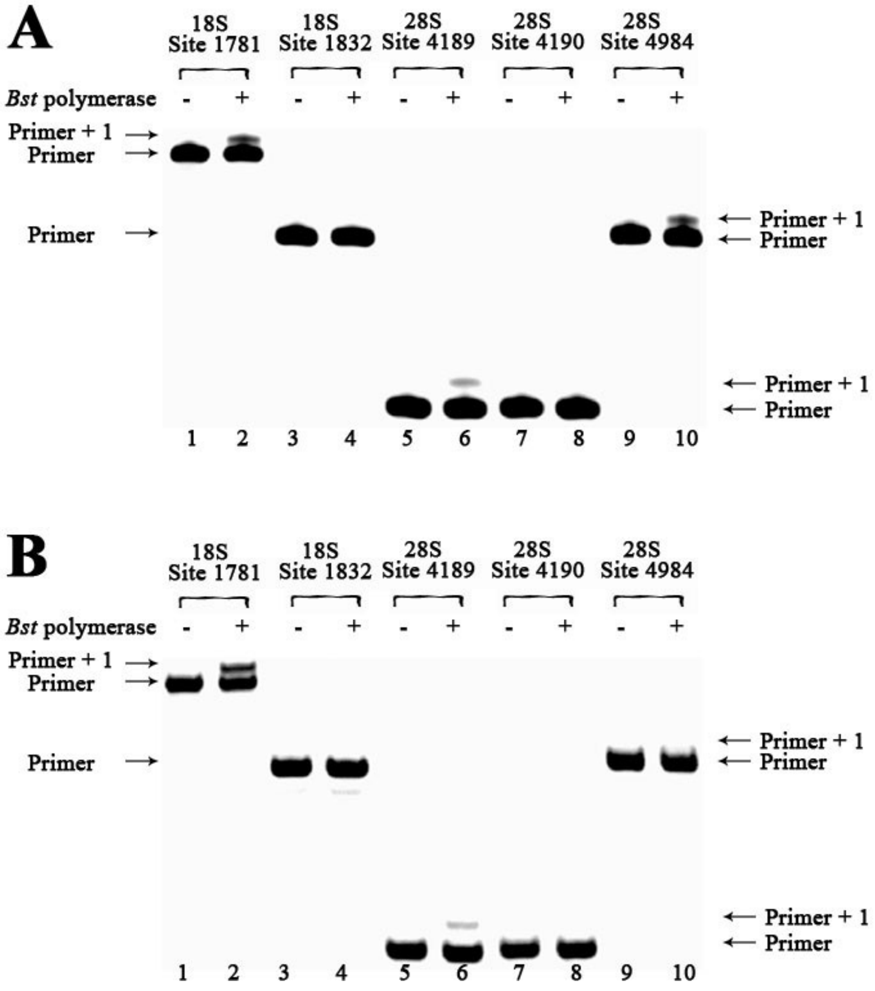

Figure S29. 18S and 28S rRNA methylation for clinical specimens was examined. 5'-FAM labelled primers were used the same as previous studies. The extension condition is as following: heating at 45 ^{o}C for 1 hour. The analysis shows the difference in extension behaviour between the studied sites. Extended DNAs were resolved on a 20% polyacrylamide denaturing gel. Lanes 1, 3, 5, 7 and 9, control samples without addition of *Bst* DNA polymerase; lanes 2, 4, 6, 8 and 10, 0.1 U *Bst* DNA polymerase was used. A) analysis for hepatic cancerous tissues of patient 3 with hepatocellular carcinoma; B) analysis for hepatic paracancerous tissues of the same patient with hepatocellular carcinoma.

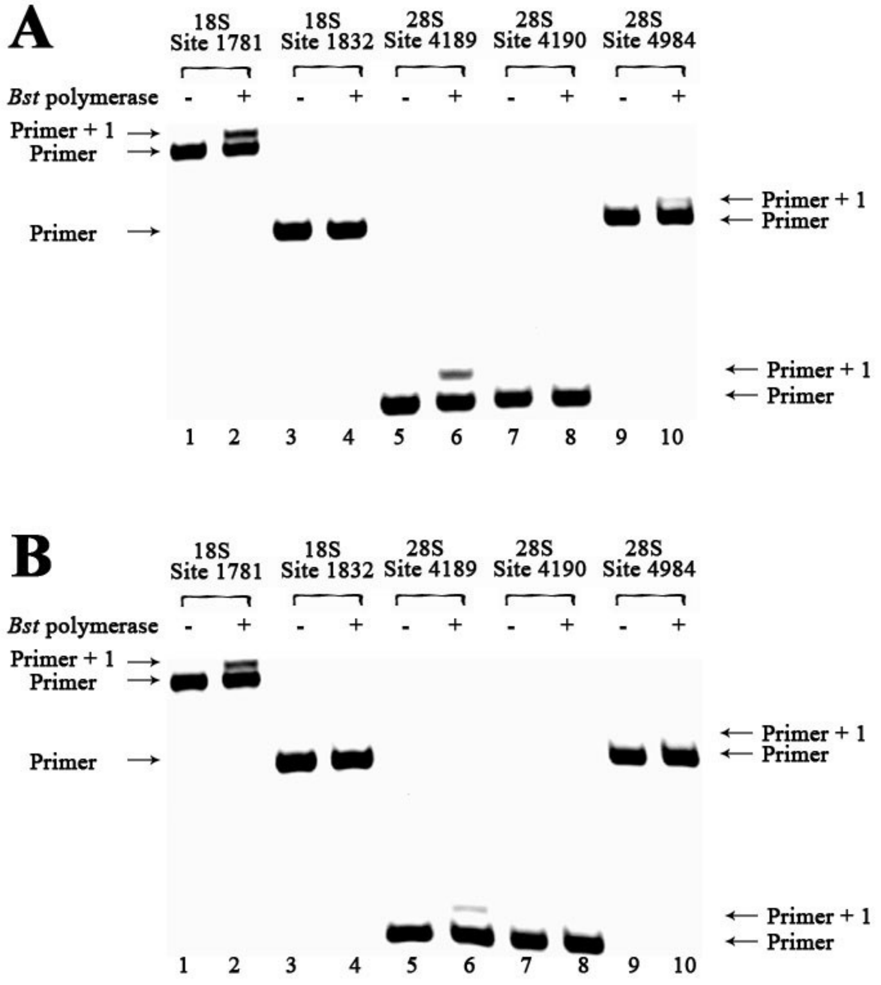

Figure S30. 18S and 28S rRNA methylation for clinical specimens was examined. 5'-FAM labelled primers were used the same as previous studies. The extension condition is as following: heating at 45 ^{o}C for 1 hour. The analysis shows the difference in extension behaviour between the studied sites. Extended DNAs were resolved on a 20% polyacrylamide denaturing gel. Lanes 1, 3, 5, 7 and 9, control samples without addition of *Bst* DNA polymerase; lanes 2, 4, 6, 8 and 10, 0.1 U *Bst* DNA polymerase was used. A) analysis for hepatic cancerous tissues of patient 4 with hepatocellular carcinoma; B) analysis for hepatic paracancerous tissues of the same patient with hepatocellular carcinoma.

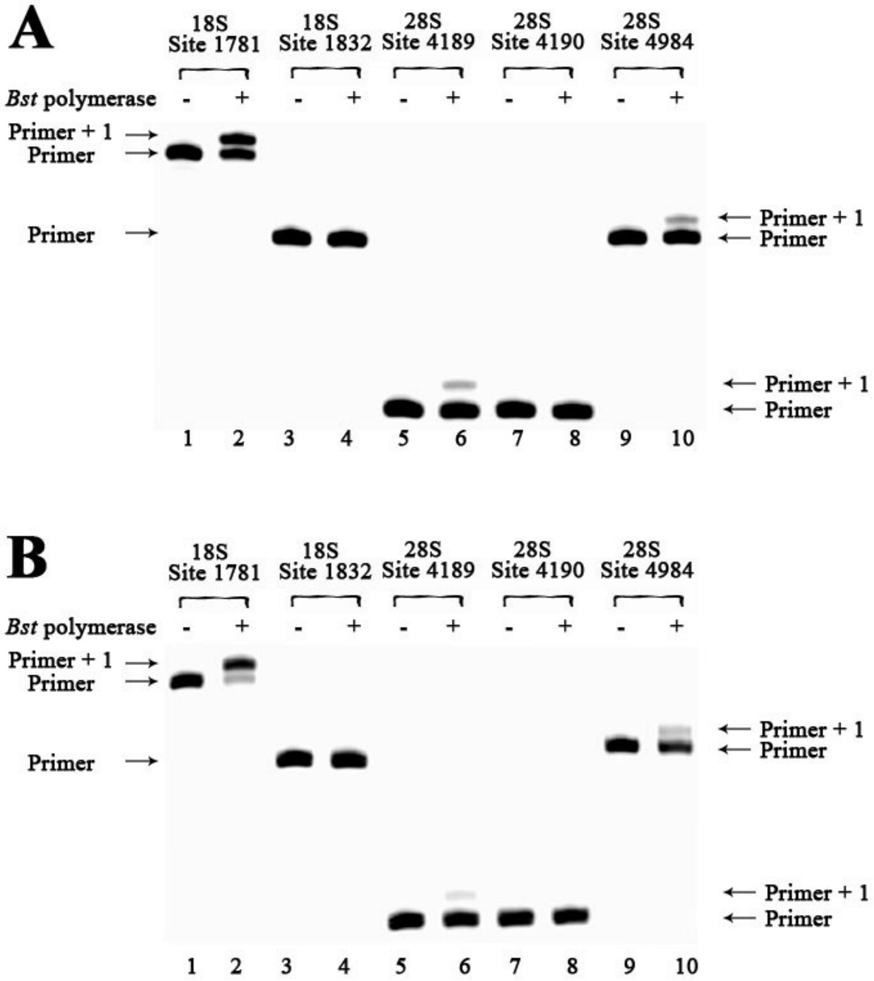

Figure S31. 18S and 28S rRNA methylation for clinical specimens was examined. 5'-FAM labelled primers were used the same as previous studies. The extension condition is as following: heating at 45 ^{o}C for 1 hour. The analysis shows the difference in extension behaviour between the studied sites. Extended DNAs were resolved on a 20% polyacrylamide denaturing gel. Lanes 1, 3, 5, 7 and 9, control samples without addition of *Bst* DNA polymerase; lanes 2, 4, 6, 8 and 10, 0.1 U *Bst* DNA polymerase was used. A) analysis for hepatic cancerous tissues of patient 5 with hepatocellular carcinoma; B) analysis for hepatic paracancerous tissues of the same patient with hepatocellular carcinoma.

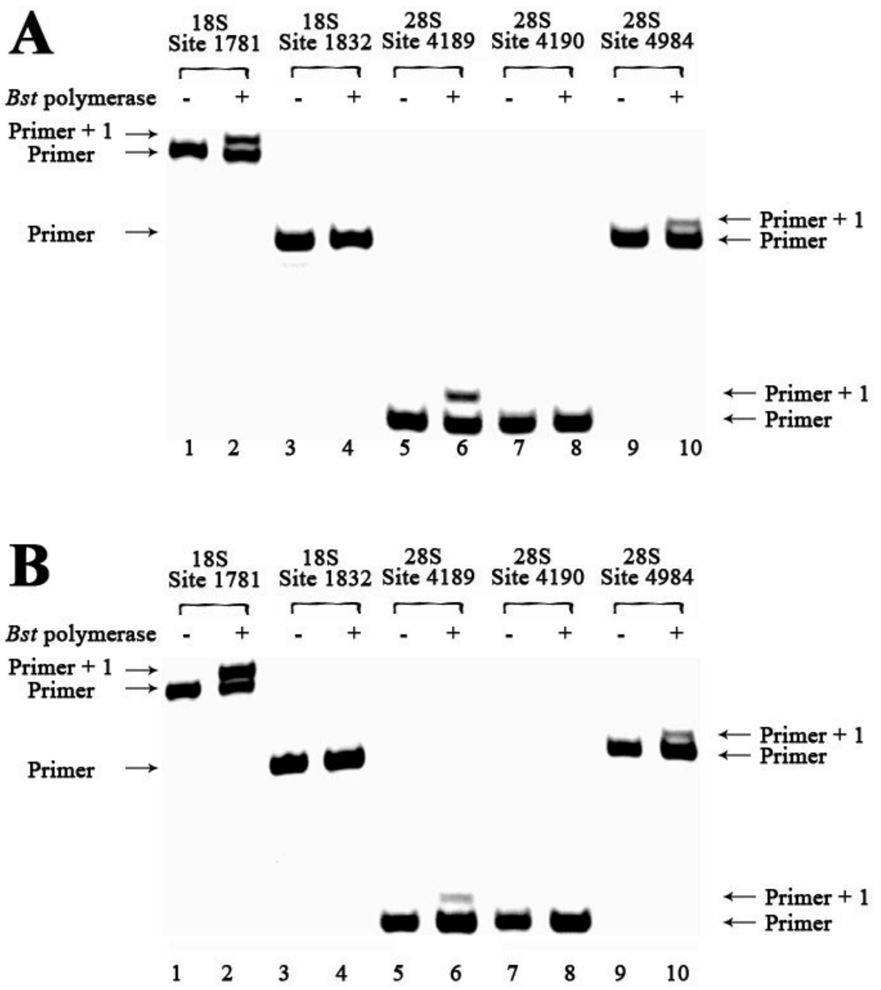

Figure S32. 18S and 28S rRNA methylation for clinical specimens was examined. 5'-FAM labelled primers were used the same as previous studies. The extension condition is as following: heating at 45 ^{o}C for 1 hour. The analysis shows the difference in extension behaviour between the studied sites. Extended DNAs were resolved on a 20% polyacrylamide denaturing gel. Lanes 1, 3, 5, 7 and 9, control samples without addition of *Bst* DNA polymerase; lanes 2, 4, 6, 8 and 10, 0.1 U *Bst* DNA polymerase was used. A) analysis for hepatic cancerous tissues of patient 6 with hepatocellular carcinoma; B) analysis for hepatic paracancerous tissues of the same patient with hepatocellular carcinoma.

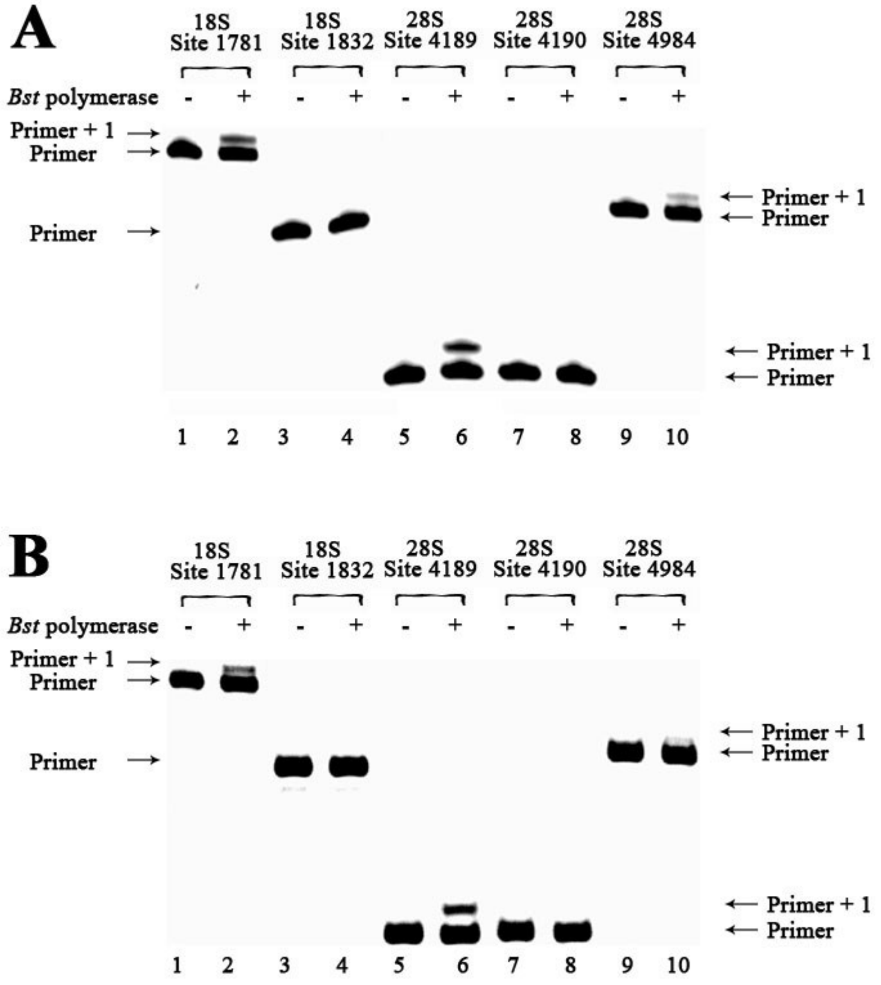

Figure S33. 18S and 28S rRNA methylation for clinical specimens was examined. 5'-FAM labelled primers were used the same as previous studies. The extension condition is as following: heating at 45 ^{o}C for 1 hour. The analysis shows the difference in extension behaviour between the studied sites. Extended DNAs were resolved on a 20% polyacrylamide denaturing gel. Lanes 1, 3, 5, 7 and 9, control samples without addition of *Bst* DNA polymerase; lanes 2, 4, 6, 8 and 10, 0.1 U *Bst* DNA polymerase was used. A) analysis for hepatic cancerous tissues of patient 7 with hepatocellular carcinoma; B) analysis for hepatic paracancerous tissues of the same patient with hepatocellular carcinoma.

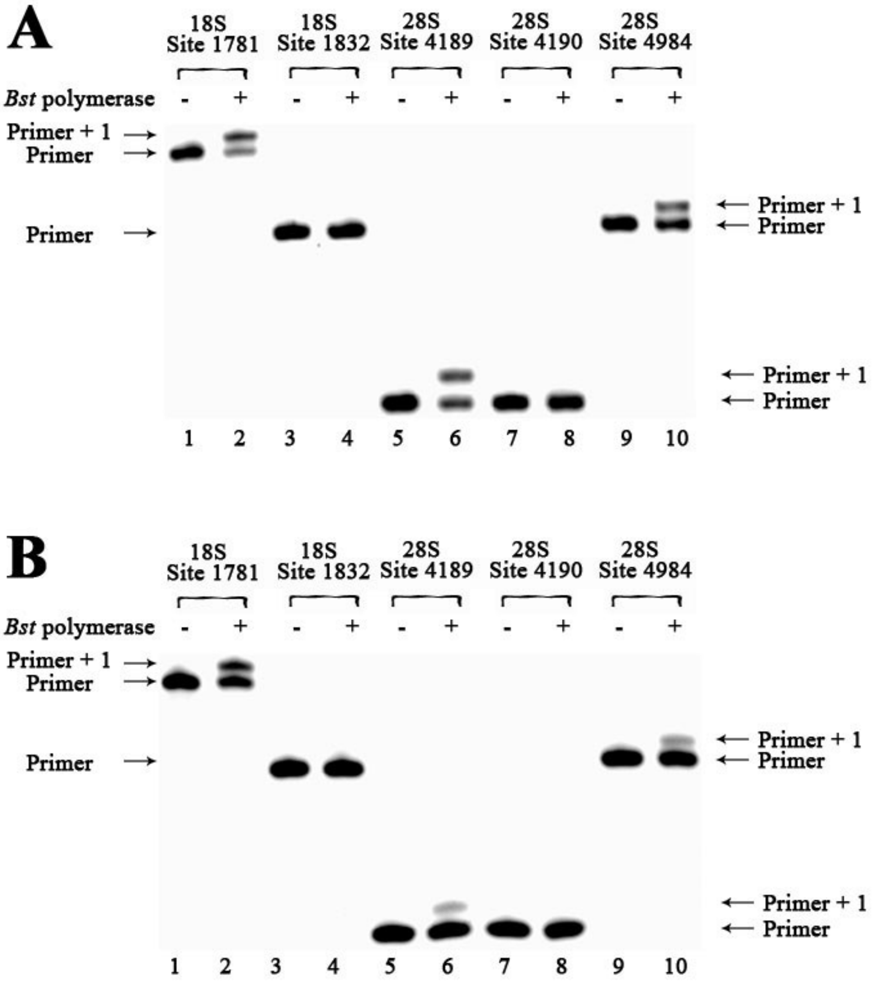

Figure S34. 18S and 28S rRNA methylation for clinical specimens was examined. 5'-FAM labelled primers were used the same as previous studies. The extension condition is as following: heating at 45 ^{o}C for 1 hour. The analysis shows the difference in extension behaviour between the studied sites. Extended DNAs were resolved on a 20% polyacrylamide denaturing gel. Lanes 1, 3, 5, 7 and 9, control samples without addition of *Bst* DNA polymerase; lanes 2, 4, 6, 8 and 10, 0.1 U *Bst* DNA polymerase was used. A) analysis for hepatic cancerous tissues of patient 8 with hepatocellular carcinoma; B) analysis for hepatic paracancerous tissues of the same patient with hepatocellular carcinoma.

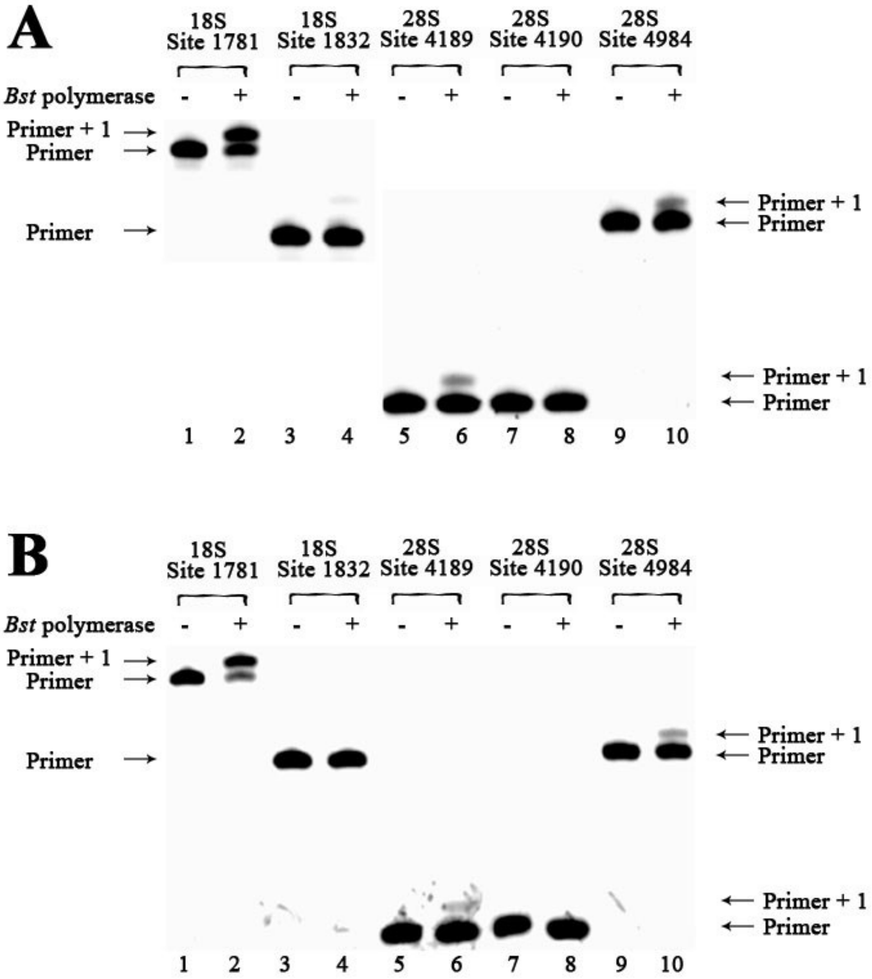

Figure S35. 18S and 28S rRNA methylation for clinical specimens was examined. 5'-FAM labelled primers were used the same as previous studies. The extension condition is as following: heating at 45 ^{o}C for 1 hour. The analysis shows the difference in extension behaviour between the studied sites. Extended DNAs were resolved on a 20% polyacrylamide denaturing gel. Lanes 1, 3, 5, 7 and 9, control samples without addition of *Bst* DNA polymerase; lanes 2, 4, 6, 8 and 10, 0.1 U *Bst* DNA polymerase was used. A) analysis for hepatic cancerous tissues of patient 9 with hepatocellular carcinoma; B) analysis for hepatic paracancerous tissues of the same patient with hepatocellular carcinoma.

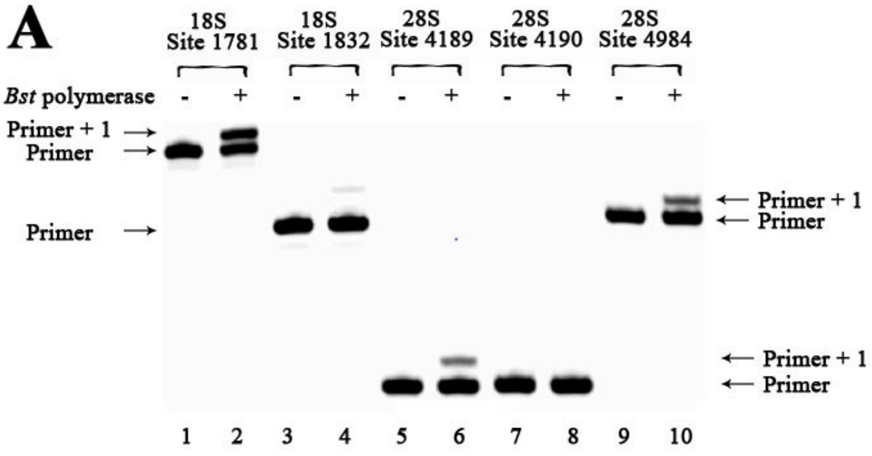

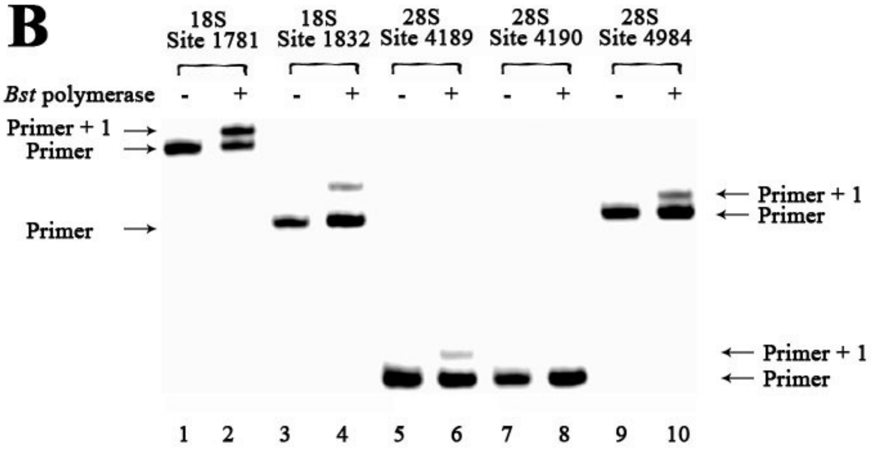

Figure S36. 18S and 28S rRNA methylation for clinical specimens was examined. 5'-FAM labelled primers were used the same as previous studies. The extension condition is as following: heating at 45 ^{o}C for 1 hour. The analysis shows the difference in extension behaviour between the studied sites. Extended DNAs were resolved on a 20% polyacrylamide denaturing gel. Lanes 1, 3, 5, 7 and 9, control samples without addition of *Bst* DNA polymerase; lanes 2, 4, 6, 8 and 10, 0.1 U *Bst* DNA polymerase was used. A) analysis for hepatic cancerous tissues of patient 10 with hepatocellular carcinoma; B) analysis for hepatic paracancerous tissues of the same patient with hepatocellular carcinoma.

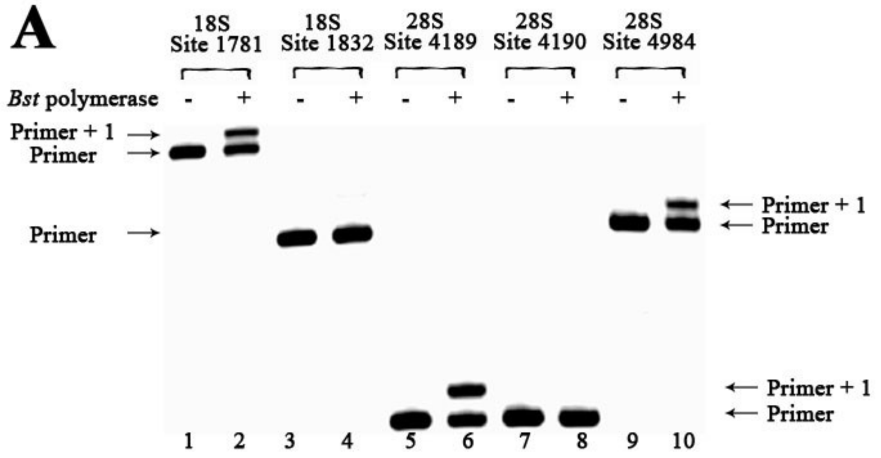

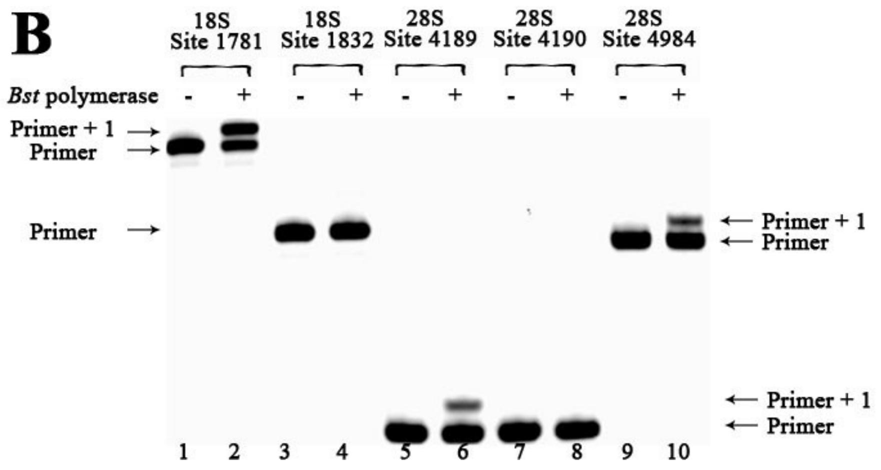

Figure S37. 18S and 28S rRNA methylation for clinical specimens was examined. 5'-FAM labelled primers were used the same as previous studies. The extension condition is as following: heating at 45 ^{o}C for 1 hour. The analysis shows the difference in extension behaviour between the studied sites. Extended DNAs were resolved on a 20% polyacrylamide denaturing gel. Lanes 1, 3, 5, 7 and 9, control samples without addition of *Bst* DNA polymerase; lanes 2, 4, 6, 8 and 10, 0.1 U *Bst* DNA polymerase was used. A) analysis for hepatic cancerous tissues of patient 11 with hepatocellular carcinoma; B) analysis for hepatic paracancerous tissues of the same patient with hepatocellular carcinoma.

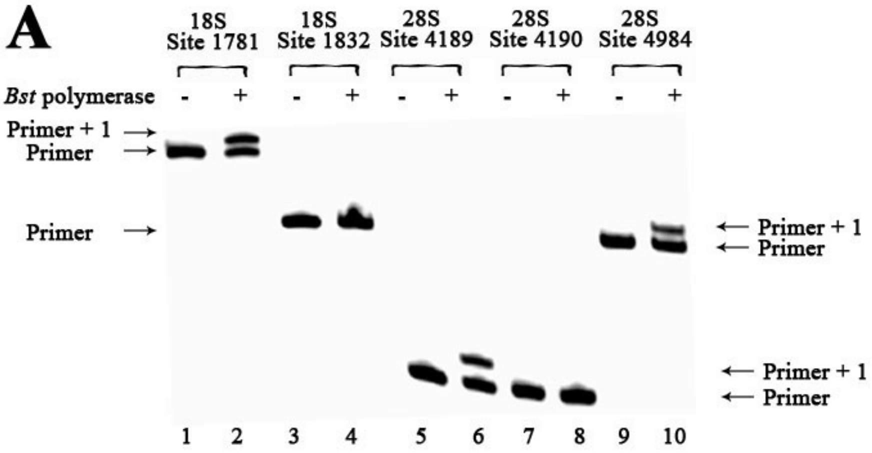

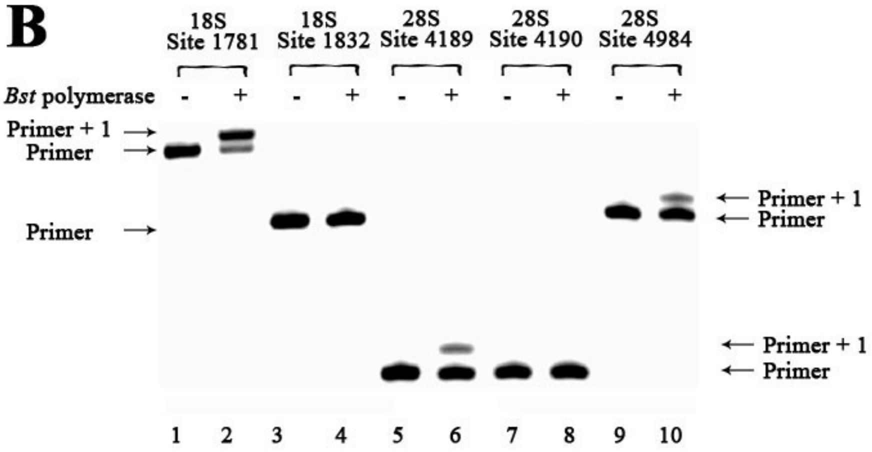

Figure S38. 18S and 28S rRNA methylation for clinical specimens was examined. 5'-FAM labelled primers were used the same as previous studies. The extension condition is as following: heating at 45 ^{o}C for 1 hour. The analysis shows the difference in extension behaviour between the studied sites. Extended DNAs were resolved on a 20% polyacrylamide denaturing gel. Lanes 1, 3, 5, 7 and 9, control samples without addition of *Bst* DNA polymerase; lanes 2, 4, 6, 8 and 10, 0.1 U *Bst* DNA polymerase was used. A) analysis for hepatic cancerous tissues of patient 12 with hepatocellular carcinoma; B) analysis for hepatic paracancerous tissues of the same patient with hepatocellular carcinoma.

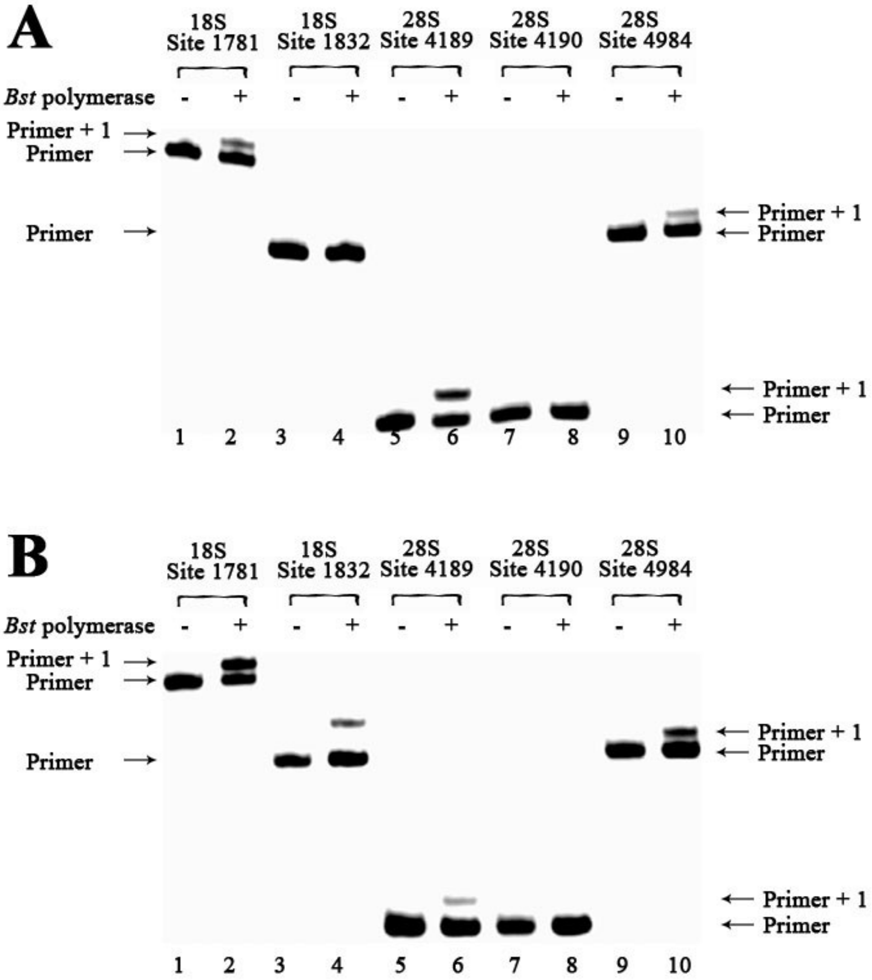

Figure S39. 18S and 28S rRNA methylation for clinical specimens was examined. 5'-FAM labelled primers were used the same as previous studies. The extension condition is as following: heating at 45 ^{o}C for 1 hour. The analysis shows the difference in extension behaviour between the studied sites. Extended DNAs were resolved on a 20% polyacrylamide denaturing gel. Lanes 1, 3, 5, 7 and 9, control samples without addition of *Bst* DNA polymerase; lanes 2, 4, 6, 8 and 10, 0.1 U *Bst* DNA polymerase was used. A) analysis for hepatic cancerous tissues of patient 13 with hepatocellular carcinoma; B) analysis for hepatic paracancerous tissues of the same patient with hepatocellular carcinoma.

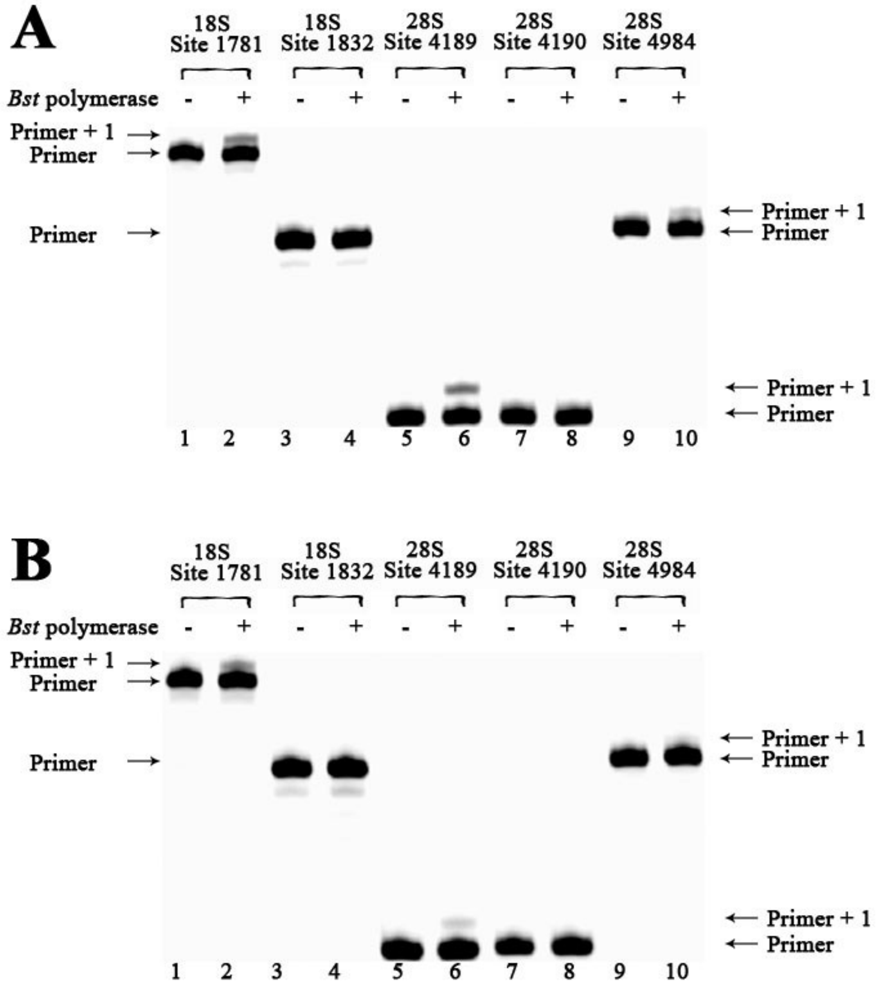

Figure S40. 18S and 28S rRNA methylation for clinical specimens was examined. 5'-FAM labelled primers were used the same as previous studies. The extension condition is as following: heating at 45 ^{o}C for 1 hour. The analysis shows the difference in extension behaviour between the studied sites. Extended DNAs were resolved on a 20% polyacrylamide denaturing gel. Lanes 1, 3, 5, 7 and 9, control samples without addition of *Bst* DNA polymerase; lanes 2, 4, 6, 8 and 10, 0.1 U *Bst* DNA polymerase was used. A) analysis for hepatic cancerous tissues of patient 14 with hepatocellular carcinoma; B) analysis for hepatic paracancerous tissues of the same patient with hepatocellular carcinoma.

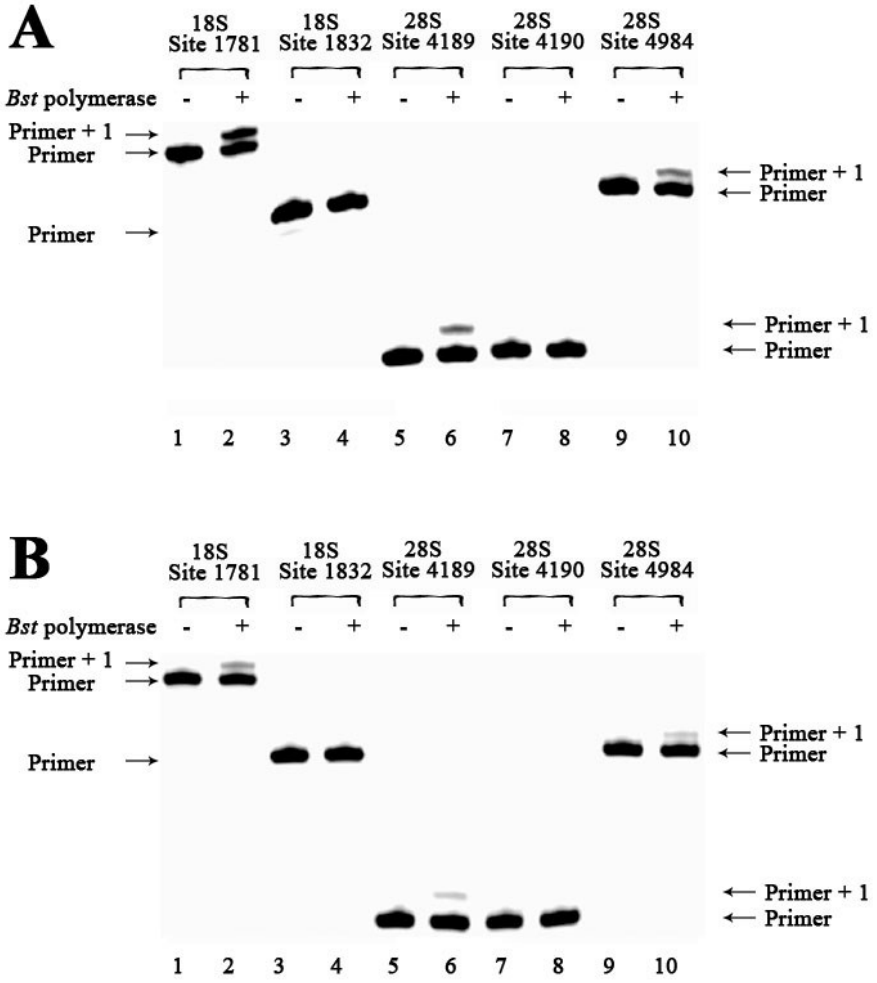

Figure S41. 18S and 28S rRNA methylation for clinical specimens was examined. 5'-FAM labelled primers were used the same as previous studies. The extension condition is as following: heating at 45 ^{o}C for 1 hour. The analysis shows the difference in extension behaviour between the studied sites. Extended DNAs were resolved on a 20% polyacrylamide denaturing gel. Lanes 1, 3, 5, 7 and 9, control samples without addition of *Bst* DNA polymerase; lanes 2, 4, 6, 8 and 10, 0.1 U *Bst* DNA polymerase was used. A) analysis for hepatic cancerous tissues of patient 15 with hepatocellular carcinoma; B) analysis for hepatic paracancerous tissues of the same patient with hepatocellular carcinoma.

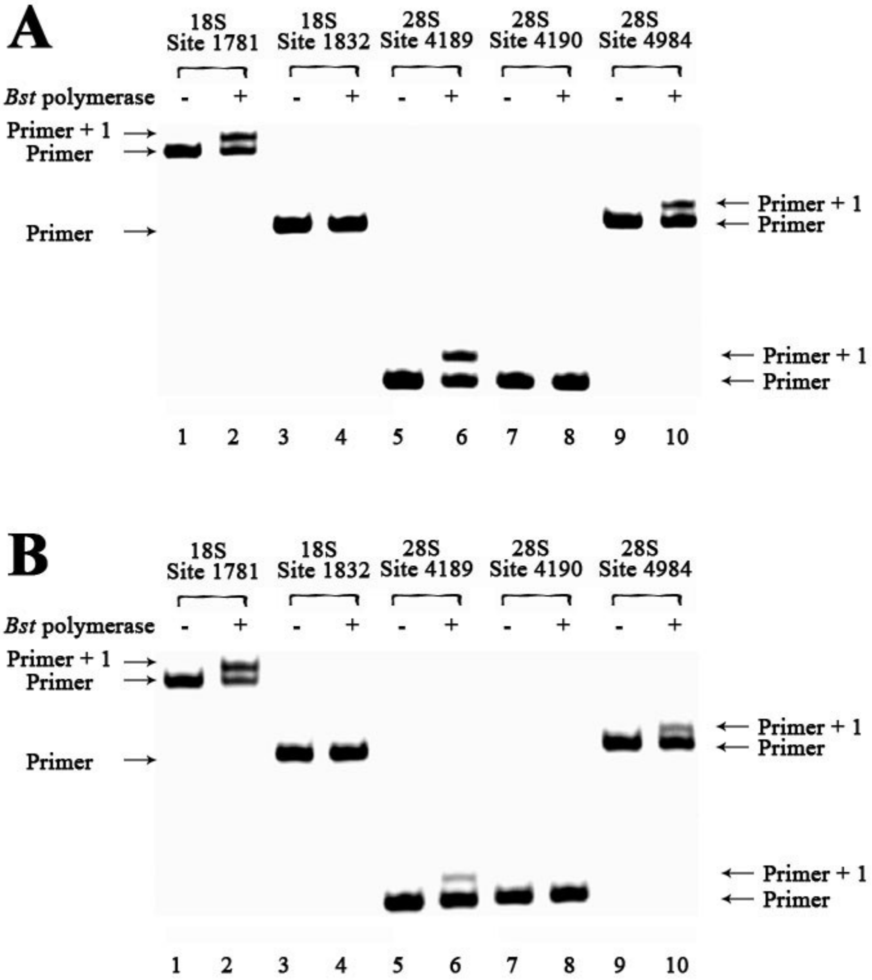

Figure S42. 18S and 28S rRNA methylation for clinical specimens was examined. 5'-FAM labelled primers were used the same as previous studies. The extension condition is as following: heating at 45 ^{o}C for 1 hour. The analysis shows the difference in extension behaviour between the studied sites. Extended DNAs were resolved on a 20% polyacrylamide denaturing gel. Lanes 1, 3, 5, 7 and 9, control samples without addition of *Bst* DNA polymerase; lanes 2, 4, 6, 8 and 10, 0.1 U *Bst* DNA polymerase was used. A) analysis for hepatic cancerous tissues of patient 16 with hepatocellular carcinoma; B) analysis for hepatic paracancerous tissues of the same patient with hepatocellular carcinoma.

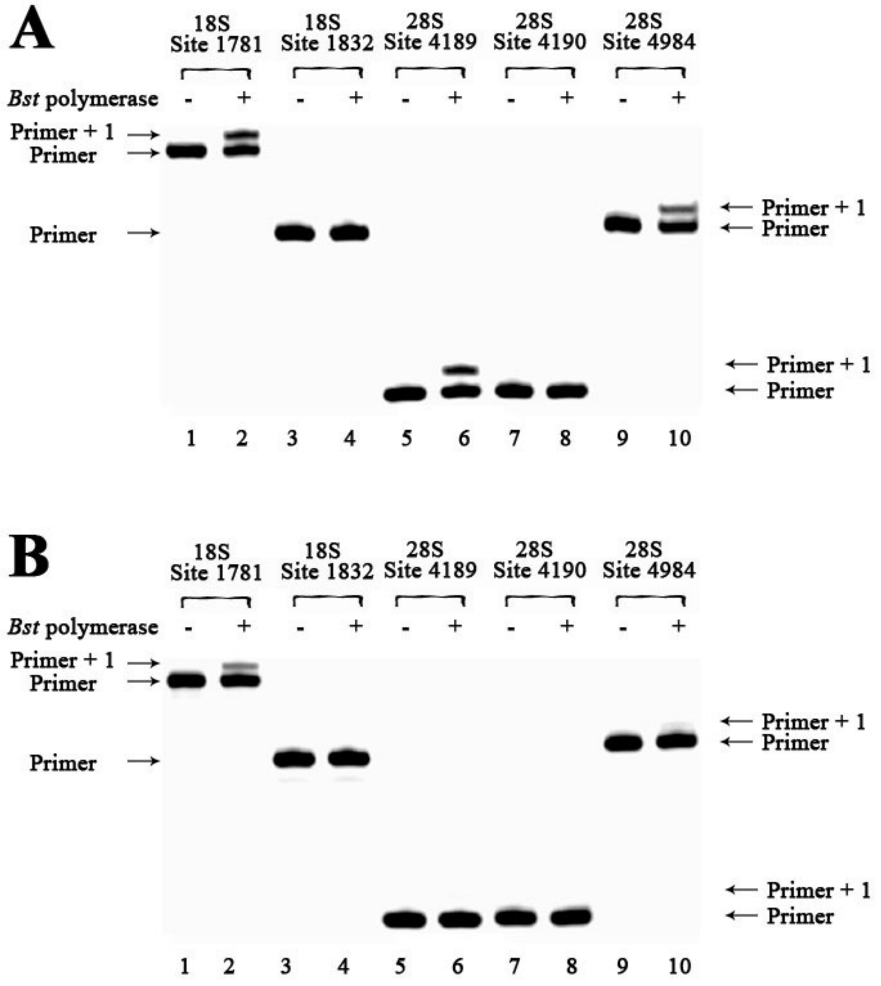

Figure S43. 18S and 28S rRNA methylation for clinical specimens was examined. 5'-FAM labelled primers were used the same as previous studies. The extension condition is as following: heating at 45 ^{o}C for 1 hour. The analysis shows the difference in extension behaviour between the studied sites. Extended DNAs were resolved on a 20% polyacrylamide denaturing gel. Lanes 1, 3, 5, 7 and 9, control samples without addition of *Bst* DNA polymerase; lanes 2, 4, 6, 8 and 10, 0.1 U *Bst* DNA polymerase was used. A) analysis for hepatic cancerous tissues of patient 17 with hepatocellular carcinoma; B) analysis for hepatic paracancerous tissues of the same patient with hepatocellular carcinoma.

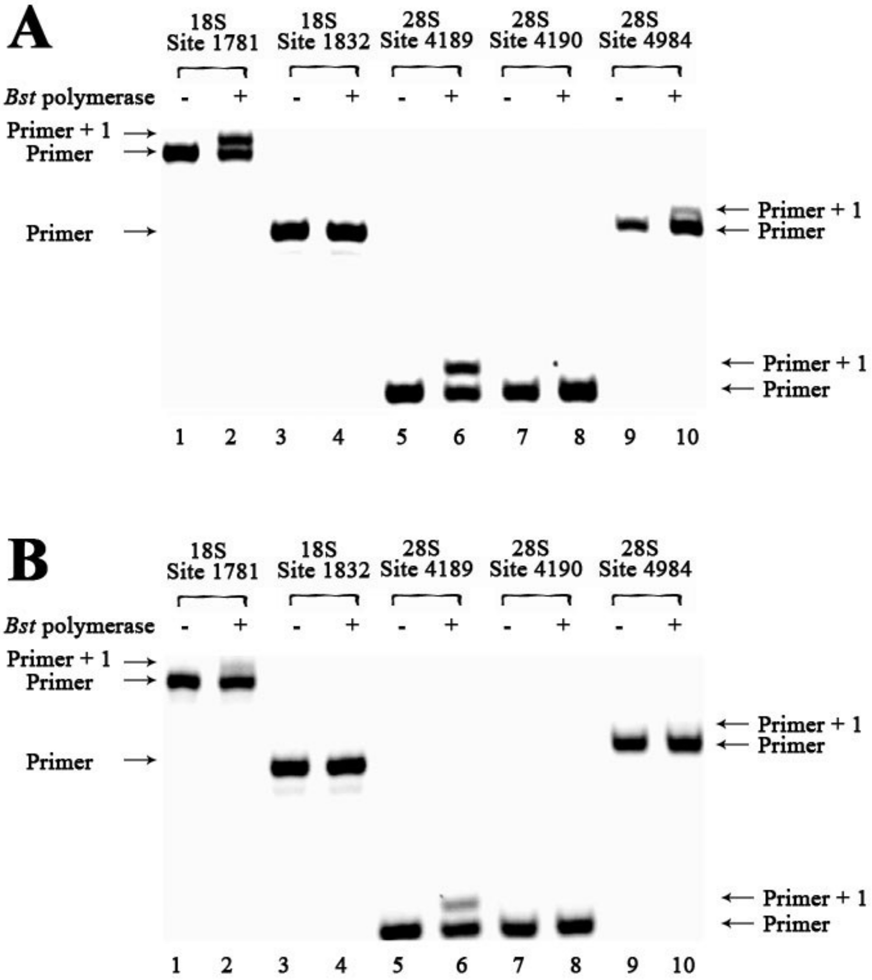

Figure S44. 18S and 28S rRNA methylation for clinical specimens was examined. 5'-FAM labelled primers were used the same as previous studies. The extension condition is as following: heating at 45 ^{o}C for 1 hour. The analysis shows the difference in extension behaviour between the studied sites. Extended DNAs were resolved on a 20% polyacrylamide denaturing gel. Lanes 1, 3, 5, 7 and 9, control samples without addition of *Bst* DNA polymerase; lanes 2, 4, 6, 8 and 10, 0.1 U *Bst* DNA polymerase was used. A) analysis for hepatic cancerous tissues of patient 18 with hepatocellular carcinoma; B) analysis for hepatic paracancerous tissues of the same patient with hepatocellular carcinoma.

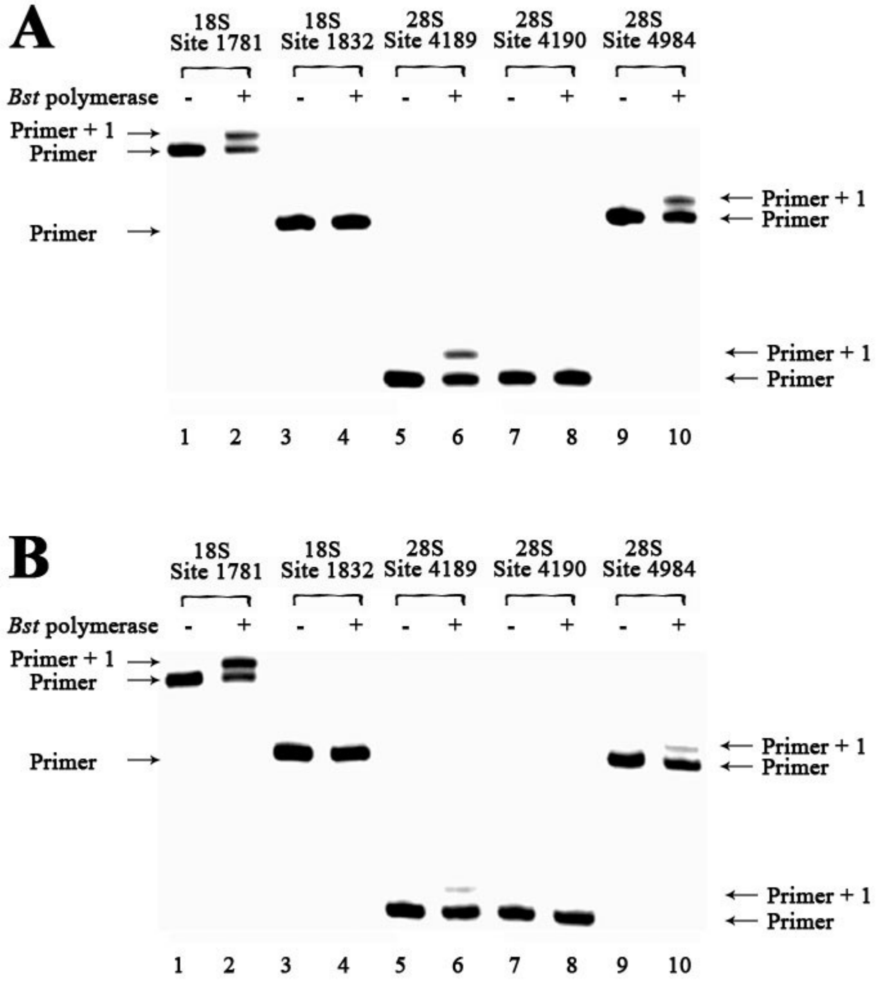

Figure S45. 18S and 28S rRNA methylation for clinical specimens was examined. 5'-FAM labelled primers were used the same as previous studies. The extension condition is as following: heating at 45 ^{o}C for 1 hour. The analysis shows the difference in extension behaviour between the studied sites. Extended DNAs were resolved on a 20% polyacrylamide denaturing gel. Lanes 1, 3, 5, 7 and 9, control samples without addition of *Bst* DNA polymerase; lanes 2, 4, 6, 8 and 10, 0.1 U *Bst* DNA polymerase was used. A) analysis for hepatic cancerous tissues of patient 19 with hepatocellular carcinoma; B) analysis for hepatic paracancerous tissues of the same patient with hepatocellular carcinoma.

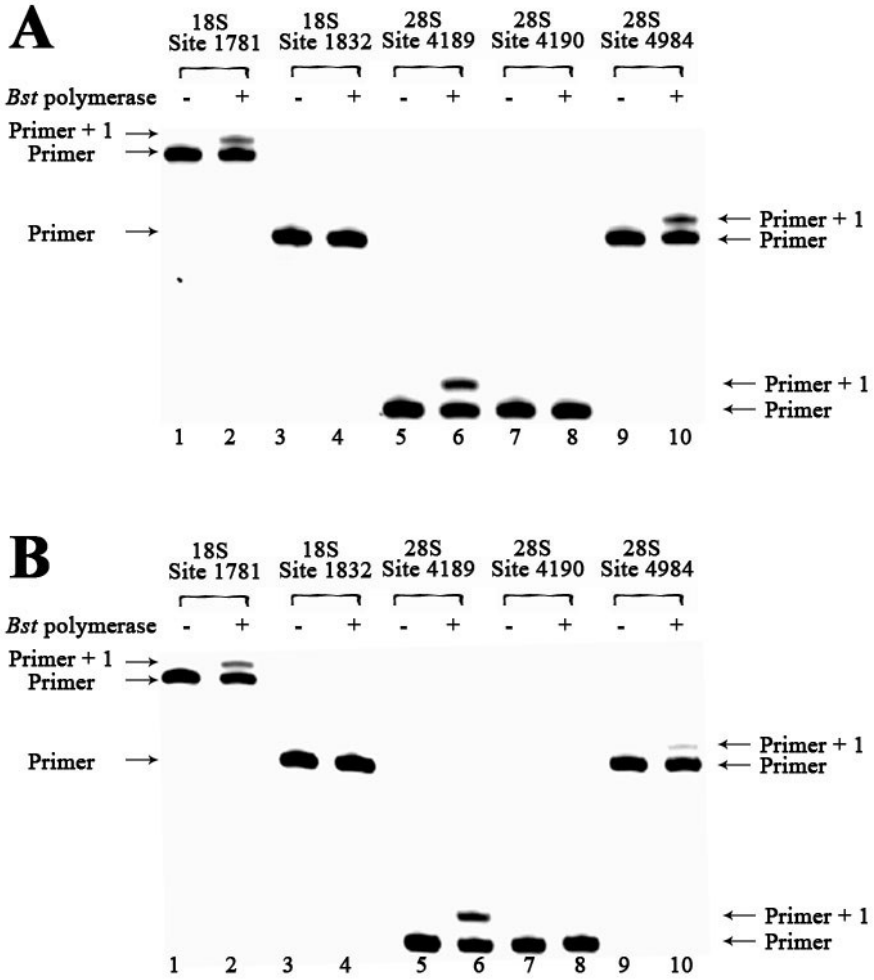

Figure S46. 18S and 28S rRNA methylation for clinical specimens was examined. 5'-FAM labelled primers were used the same as previous studies. The extension condition is as following: heating at 45 ^{o}C for 1 hour. The analysis shows the difference in extension behaviour between the studied sites. Extended DNAs were resolved on a 20% polyacrylamide denaturing gel. Lanes 1, 3, 5, 7 and 9, control samples without addition of *Bst* DNA polymerase; lanes 2, 4, 6, 8 and 10, 0.1 U *Bst* DNA polymerase was used. A) analysis for hepatic cancerous tissues of patient 20 with hepatocellular carcinoma; B) analysis for hepatic paracancerous tissues of the same patient with hepatocellular carcinoma.

References

[1] J. R. Kiefer, C. Mao, J. C. Braman and L. S. Beese, *Nature* **1998**, *391*, 304-307.

[2] E. F. Pettersen, T. D. Goddard, C. C. Huang, G. S. Couch, D. M. Greenblatt, E. C. Meng and T. E. Ferrin, *J Comput Chem* **2004**, *25*, 1605-1612.

[3] a) D. A. Case, T. E. Cheatham, 3rd, T. Darden, H. Gohlke, R. Luo, K. M. Merz, Jr., A. Onufriev, C. Simmerling, B. Wang and R. J. Woods, *J Comput Chem* **2005**, *26*, 1668-1688; b) D. Case, T. Darden, T. Cheatham III, C. Simmerling, J. Wang, R. Duke, R. Luo, R. Walker, W. Zhang and K. Merz, *University of California, San Francisco* **2012**, *1*, 3.

[4] T. E. Cheatham, 3rd, P. Cieplak and P. A. Kollman, *J Biomol Struct Dyn* **1999**, *16*, 845-862.

[5] J. Wang, R. M. Wolf, J. W. Caldwell, P. A. Kollman and D. A. Case, *J Comput Chem* **2004**, *25*, 1157-1174.

[6] E. Westhof and M. Sundaralingam, *Journal of the American Chemical Society* **1980**, *102*, 1493-1500.

[7] H. R. Drew, R. M. Wing, T. Takano, C. Broka, S. Tanaka, K. Itakura and R. E. Dickerson, *Proc Natl Acad Sci U S A* **1981**, *78*, 2179-2183.

[8] V. K. Batra, W. A. Beard, D. D. Shock, J. M. Krahn, L. C. Pedersen and S. H. Wilson, *Structure* **2006**, *14*, 757-766.
